# Under What Conditions Can Recursion Be Learned? Effects of Starting Small in Artificial Grammar Learning of Center‐Embedded Structure

**DOI:** 10.1111/cogs.12685

**Published:** 2018-09-27

**Authors:** Fenna H. Poletiek, Christopher M. Conway, Michelle R. Ellefson, Jun Lai, Bruno R. Bocanegra, Morten H. Christiansen

**Affiliations:** ^1^ Institute of Psychology Leiden University; ^2^ Max Planck Institute for Psycholinguistics Nijmegen; ^3^ Boys Town National Research Hospital Omaha Nebraska; ^4^ Faculty of Education University of Cambridge; ^5^ Erasmus School of Social and Behavioral Sciences Erasmus University; ^6^ Department of Psychology Cornell University

**Keywords:** Artificial grammar learning, Center‐embedded structures, Starting small, Statistical learning

## Abstract

It has been suggested that external and/or internal limitations paradoxically may lead to superior learning, that is, the concepts of *starting small* and *less is more* (Elman, 1993; Newport, 1990). In this paper, we explore the type of incremental ordering during training that might help learning, and what mechanism explains this facilitation. We report four artificial grammar learning experiments with human participants. In Experiments 1a and 1b we found a beneficial effect of starting small using two types of simple recursive grammars: right‐branching and center‐embedding, with recursive embedded clauses in fixed positions and fixed length. This effect was replicated in Experiment 2 (*N = *100). In Experiment 3 and 4, we used a more complex center‐embedded grammar with recursive loops in variable positions, producing strings of variable length. When participants were presented an incremental ordering of training stimuli, as in natural language, they were better able to generalize their knowledge of simple units to more complex units when the training input “grew” according to structural complexity, compared to when it “grew” according to string length. Overall, the results suggest that starting small confers an advantage for learning complex center‐embedded structures when the input is organized according to structural complexity.

## 1 Introduction

Intuitively, one would think that learners should acquire information better when they are unhindered by internal or external limitations, such as those relating to constraints on memory or input. However, some proposals take the somewhat paradoxical stance that cognitive limitations and/or reduced input may confer a computational advantage for learning. These theories, specifically the notion that *less is more* (Newport, 1990) and the importance of *starting small* (Elman, 1990, 1993), are often couched in terms of language acquisition. When learning requires discovering relationships between component elements, as is the case in language acquisition, processing limitations may be advantageous because they act as a filter to reduce memory load as well as the complexity of the problem space, making learning more manageable. The starting‐small effect is considered to be of central importance to both the fields of linguistics and developmental psychology, because it suggests that starting with a simple initial state and limited memory capacity may make it feasible to learn complex input relationships, such as those found in language, without having to postulate innate linguistic knowledge.

Unfortunately, the evidence related to starting small is far from conclusive. Children appear to learn some aspects of language better than adults; however, this result may be due to any number of factors (e.g., Hakuta, Bialystok, & Wiley, 2003; see Arnon & Christiansen, 2017, for a review). Initially, computational work supported the theory of starting small (e.g., Elman, 1993), but subsequent simulations appeared to contradict those findings (Rohde & Plaut, 1999, 2003). Furthermore, empirical data gathered from human participants have not resolved the issue; some data support a benefit for starting small (Cochran, McDonald, & Parault, 1999; Kareev, Lieberman, & Lev, 1997; Kersten & Earles, 2001; Lai & Poletiek, 2011; Poletiek, 2011), while other data do not (Fletcher, Maybery, & Bennett, 2000; Ludden & Gupta, 2000; for reviews see Rohde & Plaut, 1999, 2003).

This paper seeks to determine under what conditions, if any, starting small might have an effect on learning complex recursive language‐like structure. Specifically, we investigate the limits of the *starting small* hypothesis using the artificial grammar learning (AGL) paradigm. First, we discuss the inconclusive evidence for starting small and two possible explanations of the effect in terms of either structural complexity versus memory load. Second, we present five experiments to examine the starting‐small effect using recursive artificial grammars. In Experiment 1a and 1b, we establish the basic facilitation effect of an incremental ordering of the input for learning a grammar with multiple clauses. Experiment 1a shows that when participants are presented with a learning set generated by a simple right‐branching recursive grammar that is ordered according to the number of recursive loops at the end of strings, they achieve better learning than when the learning set is ordered randomly. Experiment 1b shows that this facilitation also occurs when the recursive loops are inserted within the string, that is, for a recursive center‐embedded (CE) grammar. In Experiment 2, the facilitation is replicated for the CE grammar using a large sample (*N = *100), and it is shown to occur both for short and long strings of the grammar.

Experiment 3 directly compares the effect of starting small according to structural complexity versus item length. The results of Experiment 3 reveal that the starting‐small effect is largest when the training set is ordered according to structural characteristics of the grammar, compared to when the ordering is according to sentence length. Finally, Experiment 4 uses a serial presentation of syllables to more closely mimic serial presentation of components in speech. The results again revealed a beneficial effect of starting small. Crucially, an additional analysis showed that only when the training set was staged according to stimulus complexity could participants use their knowledge of the simple structures to help learn the more complex ones. Our findings suggest that the facilitative effect of starting small occurs both for simple recursive structures as well as more complex ones, and it is greatest when the input “grows” incrementally according to structural complexity. In sum, these findings point to a fundamental moderator of learning that has consequences for language acquisition, development, and inductive learning more generally.

### 1.1 Starting‐small evidence

The *less is more* and *starting small* hypotheses can be thought of as two related but separate ideas. The ideas are similar in that they propose that limitations may benefit learning, but they differ in terms of the nature of the limitation that causes the benefit. The limitations may arise from internal cognitive constraints or from external constraints on the input of the cognitive system. Orthogonal to this distinction, external or internal limitations may either exert their beneficial effects on the amount or complexity of the information to be processed. As a result, the *less is more* hypothesis may refer to the benefit of internal constraints due to limited memory capacity or computational capacity (though both cognitive functions may be related, Baddeley, 2000; MacDonald & Christiansen, 2002; Poletiek, 2011). Analogously, the *starting small* hypothesis may refer to the benefit of external constraints due to limited amount of information in the input items (e.g., length) or to their limited structural complexity. Here, we review data related to all these possibilities, starting with internal constraints.

In the context of language acquisition, Newport (1990) proposed that maturational constraints in the cognitive system are crucial for allowing language to be learned successfully. In support, data were reported from deaf adult participants, who learned American Sign Language (ASL) at different ages. On ASL morphology and syntax, native signers outscored early learners, who in turn outscored late learners. Newport suggested that young children necessarily focus on smaller linguistic segments—where smaller segments refer to simpler, adjacent, structures—because of their limited working memory capacity. In this manner, children become proficient with the constituent parts of signs first and then learn to combine them into larger, more complex structures. Late learners, because they lack these cognitive limitations, attempt to learn larger and complex sequences in their entirety. Although the late learners learn quickly compared to the early learners, they are less proficient at combining simple constituents into more complex wholes.

In a subsequent study exploring this hypothesis, Elman (1993) trained a simple recurrent network (SRN) to learn aspects of an artificial language. Under standard conditions, the network was unable to learn the sequential regularities of the grammar. But when Elman simulated working memory limitations by periodically eliminating the network's access to its prior internal states—and allowing the size of this temporal window to increase over time—the neural network's performance improved.

Further support for the *less is more* hypothesis comes from Cochran et al. (1999), who taught adults a modified version of ASL. They simulated cognitive limitations by administering a concurrent capacity‐limiting task during training and found that the participants in the no‐load condition displayed more rigid learning and were less adept at using the signs in new contexts. Additionally, Kareev et al. (1997) explored the relation between working memory capacity and the detection of correlation. Human participants were tested on their ability to predict the relationship between two binary variables. Participants with lower working memory capacity were better at detecting the appropriate correlation and performed better on the task than did high working memory capacity participants. Since working memory, on this account, has both a short‐term storage and a computational cognitive function, this evidence was taken to support the hypothesis that a restricted cognitive capacity can confer an advantage in some inductive learning tasks. Finally, DeCaro, Thomas, and Beilock (2008) argued that word category structure that is learned implicitly by information integration suffers from high working memory capacity.

However, there may be reasons to be less confident in these findings. For instance, Rohde and Plaut (1999, 2003) conducted neural network simulations that contradicted Elman's (1993) results. Using the same architecture, simulation parameters, and training input, Rohde and Plaut failed to observe an advantage for reduced cognitive capacity. They also questioned a number of previous conclusions (Cochran et al., 1999; Kareev et al., 1997), arguing instead that these earlier data do not support the notion that internal limitations benefit learning. Other studies appear to support this perspective. For example, adult participants under capacity‐limiting conditions failed to show an effect of starting small in an AGL task (Ludden & Gupta, 2000). In a similar vein, younger children do not surpass older children in an implicit covariation detection task (e.g., Fletcher et al., 2000). In fact, the few studies that have examined the development of implicit pattern learning show either no differences or improvements with age (e.g., Arciuli & Simpson, 2011; Jost, Conway, Purdy, Walk, & Hendricks, 2015; Thomas et al., 2004). Finally, Tharp and Pickering (2009) disputed whether the category structure tasks used by DeCaro et al. (2008) were actually recruiting the implicit system, suggesting that the less is more effect reported might be caused by specific task demands rather than in an advantage of low working memory.

The studies reviewed so far focus mainly on the effect of internal limitations on learning. However, only a few experiments have investigated the effect of external constraints on learning. The lack of research exploring whether limiting or staging input confers a learning advantage may be partly because of the widespread belief that the language input children receive does not differ substantially from that of adults. However, as Rohde and Plaut (2003) pointed out, there is evidence that child‐directed speech tends to consist of shorter utterances and less complex sentences than adult‐directed speech (e.g., Pine, 1994; Tomasello, 2003). Therefore, it may be feasible that starting with simplified and shorter utterances provides a learning advantage, and that this may help explain children's efficiency in acquiring natural language. Here also, the evidence is mixed, however: Elman (1993) and Rohde and Plaut (1999) tested this hypothesis using neural network simulations, and obtained mixed results. In an incremental input condition, Elman first exposed the network to simple and short sequences. Afterward, complex sequences were gradually introduced to the network. The grammar used by Elman consisted of recursive rules generating center‐embedded exemplars. A starting‐small regime was implemented by presenting the network with exemplars containing an increasing number of levels of embedding. When trained in this manner, the network showed a learning advantage; however, Rohde and Plaut (1999) did not replicate this starting‐small effect in a similar computer simulation.

A few studies with human participants seem to support the idea that external constraints provide a learning advantage (Kersten & Earles, 2001; Lai & Poletiek, 2011; Lany, Gomez, & Gerken, 2007; Reber, Kassin, Lewis, & Cantor, 1980). Early work (Reber et al., 1980) found better learning of a finite‐state grammar, after exposure to a training set of items blocked together according to the paths in the grammar they follow. Kersten and Earles (2001) exposed adults to an artificial language comprising both auditory nonsense sentences and visual animated events. Some of the participants were exposed to staged input: First, single words were presented along with the animated events, then sentences composed of two words, and then finally three‐word sentences. These participants fared better on tests of their understanding of the language compared to participants who were exposed to a non‐staged random input presentation. Though Kersten and Earles view this demonstration as supporting the notion of internal limitations providing a starting‐small advantage, Rohde and Plaut (2003) note that these data show the possible benefits of using a staged input training scheme. Likewise, in the study by Lany et al. (2007) participants only acquired a complex acXbcY language in which the co‐occurring aX and bY were separated by a varying c‐element when they were first trained with a simple version of the language without the celement, that is, the aXbY structure. This result is in line with an external starting‐small advantage. Finally, Lai and Poletiek's (2011) study replicated the beneficial effect of a starting‐small regimen found by Elman (1993) using an artificial center‐embedded grammar that gradually increased in complexity. Though Lai and Poletiek found a strong facilitation of starting small, the center‐embedded structure they used was quite simple compared to Elman's more naturalistic stimuli. Moreover, superficial phonological cues may have provided information about the underlying recursive dependencies in the grammar, independently of the recursive center‐embedded structure itself, likely making it easier to learn than center‐embedded constructions without such additional cues.

To sum up, we note three crucial observations. First, some empirical studies suggest that internal cognitive constraints can provide an advantage for learning, although simulation studies are inconclusive in this regard. Moreover, since memory and computation are closely intertwined cognitive functions, it is still unclear which of the two aspects—length versus computational load—is responsible for the learning advantage. Second, a few studies show that external constraints—that is, gradually increasing the complexity or quantity of information in the input—may enhance learning as well. However, in these studies (Kersten & Earles, 2001; Lai & Poletiek, 2011; Reber et al., 1980), it is also unclear which external constraints (staging the input based on complexity or length) crucially affects the learnability of the underlying structure, because manipulations of stimulus complexity often covary with stimulus length. Third, it is possible that the type of structures used to test the starting‐small effect may affect the outcome. Recursive center‐embedded structures are complex due to their long‐distance dependencies. Alternately, other types of recursive grammars are more linear in nature, adding recursive clauses at the end of strings, as in right‐branching recursion. Even simpler grammars are finite‐state grammars, as used in early AGL studies (Reber et al., 1980). One of the major successful tests of the starting‐small effect used a complex recursive structure (Elman, 1993), whereas one of the “unsuccessful” tests used a simple standard finite‐state grammar (e.g., Ludden & Gupta, 2000, Experiment II). Thus, it is possible that the advantage of starting small depends partly on the underlying structure to be learned.

Here, we explore how starting small may specifically facilitate the learning of artificial grammars that contain recursive constructions. We suggest that the learning of a particular recursive structure involves two parts: (a) learning the structural regularities defining the construction in its basic (non‐recursive) form, and (b) learning to generalize these regularities to complex recursive structures incorporating multiple instances of this construction. Note that in our view, what it means to “learn recursive structure” is to learn simple “chunks” (i.e., bigrams and trigrams) first, and then learn to recognize that the chunks can be combined into more complex structures that may involve non‐adjacent dependencies (see also Christiansen & MacDonald, 2009; Poletiek, 2011). Given that, in this view, mastery of the basic regularities is necessary for successful processing of sequences with recursive embeddings, the time course of the learning process may be crucial. Hence, presenting the input in a starting‐small fashion with additional recursive generalizations at each subsequent stage, may optimally support learning. This possibility is particularly interesting in light of the recent ongoing debate about the cognitive mechanisms supporting the acquisition of recursion in natural language, and the role of the stimulus input in this learning process (e.g., Chomsky, 1995; Christiansen & Chater, 1999, 2015; Christiansen & MacDonald, 2009; Corballis, 2007; De Vries, Christiansen, & Petersson, 2011; Fitch & Hauser, 2004; Gibson & Thomas, 1999; Oettl, Jaeger, & Kaup, 2015; Perfors, Tenenbaum, & Regier, 2011). Before presenting the four experiments that explore this hypothesis, we briefly describe the types of recursive grammars used in the present methodology.

### 1.2 Recursive artificial grammars

A recursive grammatical construction is one that is defined by self‐reference. Different types of recursion can be found across a variety of natural linguistic structures. As the number of self‐references (alternatively called recursive loops or order of complexity) increases within a recursive construction, the number of embeddings increases. Consider the grammatical English noun‐phrases in (1):

**Table:** 

(1)	a) *The dog* [*on the sidewalk*].
	b) *The dog* [*on the sidewalk*] [*near the tree*].
	c) *The dog* [*on the sidewalk*] [*near the tree*] [*by the house*].

The above sentences display *right‐branching* recursion, in which new prepositional phrases are recursively added onto the right end, creating sentences of potentially infinite length. Sentence (1a) comprises 0 level of embedding (LoE), (1b) 1‐LoE, and (1c) 2‐LoE.

Increasing the number of right‐branching embeddings results in slightly decreased comprehensibility of English sentences (Christiansen & MacDonald, 2009). Decreases in comprehension are even larger for a second type of recursive structure: *center‐embedding* (e.g., Bach, Brown, & Marslen‐Wilson, 1986). Center‐embedded recursion generates structure by embedding new material in the center, and pushing apart elements that depend on each other, resulting in long‐distance dependencies. For example, consider the sentences in (2):

**Table:** 

(2)	a) [*The boy likes the dog]*.
	b) [*The boy* [*the girl loves*] *likes the dog]*.
	c) [*The boy* [*the girl* [*the woman admires*] *loves*] *likes the dog]*.

As before, sentence (2a) comprises 0‐LoE, (2b) 1‐LoE, and (2c) 2‐LoE.

The same semantic relationships can be expressed using either right‐branching or center‐embedding recursion. For example, consider the two sentences (without recursive embeddings) having the same basic structure (3a and 3b), below. These two sentences can be combined either using right‐branching embedding as in (3c) or center‐embedding as in (3d):

**Table:** 

(3)	a) [*The boy likes the dog*].
	b) [*The girl loves the boy*].
	c) [*The girl loves the boy*] [*who likes the dog*].
	d) [*The boy* [*whom the girl loves*] *likes the dog*].

Both sentences express similar semantic content and involve equal lengthening of the sequence, though the center‐embedding construction is presumably more difficult than the right‐branching version because it involves long distance dependencies. Thus, whereas both sentences (3c) and (3d) are of the same length, they appear to differ in terms of complexity. Thus, by studying performance on right‐branching and center‐embedded stimuli, after a starting small and a random training, it may be possible to test the effect of starting small on two types of recursive grammars.

**Figure 1:**
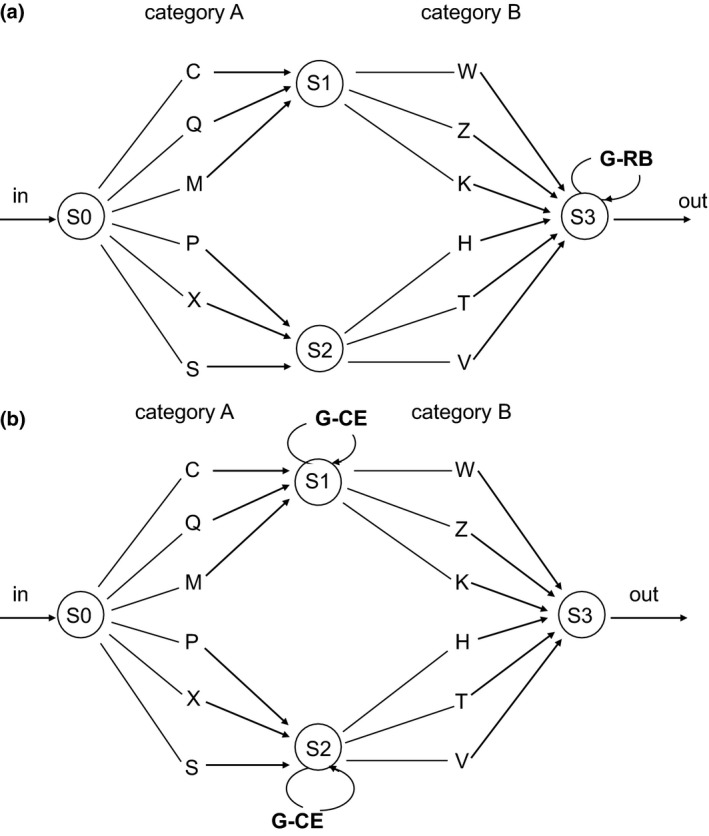
(a) Right‐branching grammar (G‐RB) used in Experiment 1a. Examples of strings generated by G‐RB are CZ, MZ[PH], and MK[PT][XT]. (b) Center‐embedding grammar (G‐CE) used in Experiments 1b and 2. Examples of strings generated by G‐CE are CZ, M[PH]Z, and M[P[XT]T]K.

Translating this question into a controlled experimental situation, we first constructed a right‐branching (Experiment 1a) and a matched center‐embedding grammar (Experiment 1b), to test the effect of a starting‐small exposure (i.e., gradually increasing the number of embeddings in the input) on learning these two types of grammars. The effect of starting small on learning center‐embedded is replicated in Experiment 2 in a larger and more diverse sample of participants. In Experiments 3 and 4, we more directly explored the separate contributions of constraints on length versus constraints on computational load in terms of leading to a learning advantage. To generate sequences of elements used in Experiments 1a, 1b, and 2, we created two categories of elements. Elements were represented as letters: Category A and Category B. Category A letters could be paired to Category B letters. The first letter from the pair belonged to Category A and the second letter of the pair belonged to Category B. Furthermore, we included two subsets within each category: Subset 1 and Subset 2. Translating this grouping in natural language syntactical categories, Category A elements might be thought of as nouns, and Category B letters as verbs. Moreover, Subset 1 and Subset 2 elements might represent singular and plural items, respectively. Accordingly, letters from Category A—Subset 1 could be paired only with letters from Category B—Subset 1. Similarly, letters from Category A—Subset 2 could be paired only with letters from Category B—Subset 2. Twelve consonants, C, Q, M, P, X, S, W, Z, K, H, T, and V, represented the subsets within each category. A recursive rule was used to generate self‐embedded exemplars. The embeddings were either right‐branching (added at the end of the exemplar) or center‐embedded (inserted in the exemplar), depending on the type of grammar, as indicated in Fig. 1a and b, respectively.

In Fig. 1a, one of the two paths starting from S3 represents the recursive loop generating a right‐branching clause. The other path from S3 terminates the string. In Fig. 1b, one of the two paths from S1 and S2 represents the recursive loop generating a center‐embedded clause. For an example of how these grammars generate recursive input strings, C[PH]W was produced from the grammar of Fig. 1b, having one level of center embedding. CW[PH][QZ] was produced from the grammar of Fig. 1a, having two levels of right‐branching embedding.

The purpose of Experiment 1a and 1b was to establish the basic facilitation effect of an incremental training regimen for learning recursive complex grammars with multiple clauses. Considering that recursive sentences are made by combining multiple building blocks with the same structure, we predict a learning regime that allows for encoding the short building blocks separately first, followed by longer sequences embodying more of these blocks, should help to make sense of the grammar. The basic facilitation was expected to show up in a controlled study with a relatively small sample. Notice that though grammar complexity can be defined in various ways (in terms of entropy, for example, see Van den Bos & Poletiek, 2008), grammar complexity and string complexity are defined here in terms of the levels of recursion it (can) contain(s). A string generated by a complex grammar is more complex as it embodies more levels of embedded loops; a simple string having no levels of embedding. To first explore the hypothesis that starting small helps with learning right‐branching recursion, we conducted Experiment 1a, testing whether a simple recursive grammar will produce the starting‐small effect when exemplars are ordered according to increasing numbers of levels of embedding and string length. The relatively small sample size of participants in the two‐first experiments was chosen on the consideration that, if starting small helps to a reasonable extent, the effect should show up in these relatively easy versions of the RB and CE structure learning tasks.

## 2 Experiment 1a

In the first experiment, we generated letter strings from an artificial grammar having right‐branching recursion (Fig. 1a). We ordered the exemplars differently for two separate training conditions. In the starting‐small condition, exemplars were ordered according to increasing levels of embedding (LoE). This corresponded to first presenting strings with 0‐LoE, then strings with 1‐LoE, and finally strings with 2‐LoE. In this way, the input “started small” with basic sequences only and progressively became more complex with applications of the right‐branching rule. In the second training condition, participants received the same input but presented in random order. We predicted that by ordering the strings in this way, the starting‐small input group would learn the basic structure of the input first and then be able to generalize it to more complex recursive structures, providing an advantage over the random group, which is exposed to both the basic and the recursive constructions in an intermixed fashion.

Notice that the present experiments do not focus on whether the learning process is implicit or explicit (Dienes & Seth, 2010). However, since some previous studies on the implicit or explicit nature of the learning process in the AGL paradigm suggest that complex grammars might benefit from an implicit learning strategy (Berry & Dienes, 1993; Reber, 1976, 1993; Reber et al., 1980; Van den Bos & Poletiek, 2008) and since the grammars we used have recursive complexity, we gave participants “implicit” instructions in all experiments and all conditions; the learning task was presented as a memory experiment, and no rules were mentioned until the test phase (as is typical in many AGL experiments).

### 2.1 Method

#### 2.1.1 Participants

For Experiment 1a, 14 undergraduate participants (7 in each condition) were recruited from psychology classes at Cornell University, earning course credits. Please note that Experiments 1a and 1b were meant to provide an initial proof of concept of the beneficial effect of starting‐small training regimes on learning recursive structure. Although we did not perform a formal power analysis, we expected these effects to be large and therefore tested relatively small samples (see Conway, Ellefson, & Christiansen, 2003).

#### 2.1.2 Materials

The stimuli were letter sequences generated from the artificial grammar displayed in Fig. 1a (see Online Appendix A). The sequences were based on the repetition of pairs, within a recursive structure, in which arbitrary letters were assigned to Subset 1 and Subset 2, and to Category A and Category B (see Fig. 1a). An example of a 0‐LoE sequence is CW, a 1‐LoE sequence is CWPT, and a 2‐LoE sequence is CWPTQZ.

Unique sequences were created for the training and test sessions. Fifty sequences comprised the training session. Of these 50 training sequences, 10 were 0‐LoE embedding, 20 were 1‐LoE embedding, and 20 were 2‐LoE. An additional 50 sequences comprised the test session (see Online Appendix A). Of these test sequences, 25 were generated from the same grammar as the training sequences (grammatical) and 25 did not follow the grammar (ungrammatical). Ungrammatical sequences were created by changing one letter of a grammatical test sequence. The substituted letter was one that was of the proper noun–verb category but with an incorrect plurality (subset). Hence, since the error involved the relative position of the “verb” with regard to its corresponding noun (the nouns could never be wrongly positioned), a learner will not be able to notice an ungrammaticality unless he or she knows the grammatical pattern of correspondences between the nouns and verbs. For example, the test item **CV**MZ had two clauses: CV and MZ. Since (the noun) C could not be followed by (the verb) V, the item was ungrammatical. The positions in which the substituted letters occurred in the sequences were distributed evenly across all items. The test session comprised 16 sequences of 0‐LoE, 16 of 1‐LoE, and 18 of 2‐LoE, with each level of embedding having half grammatical and half ungrammatical structures.

#### 2.1.3 Procedure

The experiments were run using the E‐Prime presentation software with stimuli presented on a computer monitor. Participants were randomly assigned to one of two conditions: Starting Small or Random. All participants were instructed that they were participating in a memory experiment. They were told that in the first part of the experiment they would see sequences of letters displayed on the screen and that they would be tested later on what they observed. Each sequence in its entirety was presented individually, for a duration of 4‐s each. Each of the 50 training items was presented three times, for a total of 150 input exposures, lasting roughly 12–14 min in duration. The starting‐small participants received staged input: three blocks of the 0‐LoE sequences were presented first; next three blocks of the 1‐LoE sequences, and finally three blocks of the 2‐LoE sequences. No breaks were provided between blocks. Sequences were randomized within blocks. The random group received all the sequences across all LoE intermixed with one another, in random order. Thus, both the starting small and the random groups received the same training input but in different orders of presentation.

After the training phase, participants were told that the items they had just seen had been generated by a complex set of rules that determined the order of the letters. They were instructed that they would now see new letter strings, some of which followed the rules of the grammar, and some of which did not. Their task was to classify whether each letter string followed the same rules as the training sequences or not, by pressing a button marked “YES” or “NO.” Each test sequence was presented on the screen until a judgment was made, with no time limit given. Both the starting‐small and random groups received the same test instructions and the same set of 50 test sequences were presented in random order for each participant.

### 2.2 Results and discussion

**Figure 2:**
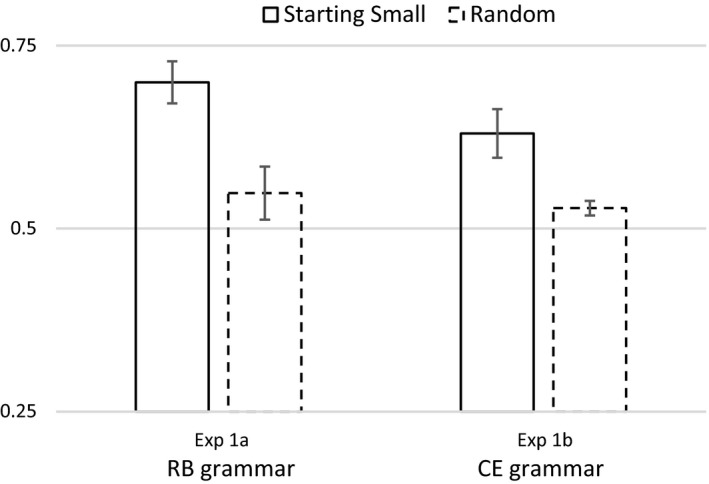
Performance for Starting Small and Random‐ordering training conditions using a right‐branching (Experiment 1a) and a center‐embedded recursive grammar (Experiment 1b). Error bars indicate *SE* of the mean.

The mean proportion of correct classification of the 50 test items was .70 for the starting‐small group (*M = *35.00, *SD* = 3.79) and .54 for the random group (*M = *27.43, *SD* = 4.79). We conducted one‐sample *t* tests and found that only the starting‐small group performed significantly above chance levels (*t*(6) = 6.99, *p *<* *.001). The starting‐small group also performed significantly better than the random group (*t*(12) = 3.86, *p *<* *.01; see Fig. 2).

The results of Experiment 1a show that only when the input was presented in a staged fashion, with 0‐LoE strings presented first, did participants show above‐chance learning of the artificial grammar. Participants showed no learning when the training items were presented in random order. Crucially, the starting‐small group out‐performed the random group, lending empirical support to the starting small hypothesis.

## 3 Experiment 1b

In Experiment 1a, we observed an effect of starting‐small for a right‐branching recursive structure. This involves the addition of new basic 0‐LoE construction at the end of a grammatical sequence. In the resulting grammatical sequence, the grammatical dependencies are all between adjacent elements in a string. We next explore to what extent the starting‐small effect is also present in center‐embedding recursion, which is characterized by *non‐adjacent* dependencies (Fig. 1b); here, the basic 0‐LoE structure has to be recognized even if the two connected elements it is made of (an A category and a B category letter) are pulled apart to distant positions.

We predicted that by ordering the strings, the starting‐small input group would be able to generalize the basic agreement structure from the 0‐LoE items to the more complex center‐embedded constructions. In contrast, the random group was expected to have problems learning this grammar as they were presented with both basic and recursive items intermixed with one another. However, as the center‐embedded operation is more complex, lower performance is expected than for the right‐branching structure, when participants are provided with the same number of learning items as in Experiment 1a.

### 3.1 Method

#### 3.1.1 Participants

For Experiment 1b, 16 undergraduate participants (8 in each condition) were recruited from psychology classes at Cornell University, earning extra credit.

#### 3.1.2 Materials

The sequences used in Experiment 1b were identical to those in Experiment 1a except that they were converted from a right‐branching to a center‐embedded structure (see Online Appendix B). That is, embedding was increased by inserting additional noun–verb pairs into the middle of the center‐embedded sequences to achieve higher levels of embedding. An example of a 0‐LoE center‐embedded sequence is CW, a 1‐LoE sequence is CPTW, and a 2‐LoE sequence is CPQZTW. Test items were made in the same manner as for Experiment 1a. For example, the test item **C**MZ**V** had two clauses: CV and MZ. Since (the noun) C could not be followed by (the verb) V, the item was ungrammatical.

#### 3.1.3 Procedure

The procedure was identical to Experiment 1a.

### 3.2 Results and discussion

The mean proportion of correct classification on the 50 test items was .63 for the starting‐small group (*M = *31.5, *SD* = 4.71) and .52 for the random group (*M = *26.4, *SD* = 1.06). Only the starting‐small group performed significantly above chance levels (*t*(7) = 4.08, *p *<* *.005). The starting‐small group also performed significantly better than the random group (*t*(14) = 2.88, *p *<* *.05; see Fig. 2). In order to test for a possible interaction between type of grammar (RB vs. CE) and training regime (Starting‐small vs. random), we performed a 2 × 2 between‐subjects anova on the aggregated data of Experiments 1a and 1b. Starting small showed a beneficial effect on learning compared to a random ordering (*F*(1, 26) = 20.2, *p *<* *.001), comparing RB to CE learning (Experiment 1a with Experiment 1b), we observed no main effect of grammar (*F = *2.6, *p = *.12), nor any interaction between grammar and training regime (*F *<* *1, *p = *.63).

Though our findings clearly suggest a reliable effect of Starting Small, our sample of participants was small and homogeneous, containing educated young people only. If the starting‐small effect plays a role in natural language learning, we can expect it to underlie the acquisition of sequential regularities in the general population of learners, disregarding variation in their cognitive capacity. To explore further the relevance of the Starting Small effect and to corroborate our finding, we subsequently conducted a replication study of Experiment 1b, in a larger, more diverse sample (*N = *100).

## 4 Experiment 2

We replicated Experiment 1b using a larger number of participants sampled from a more heterogeneous population recruited using Amazon Mechanical Turk.

### 4.1 Method

#### 4.1.1 Participants

The participants were recruited using Amazon Mechanical Turk (https://www.mturk.com). Internet‐based experimentation was chosen due to the simple nature of the experiment and the large number of participants that we desired. Previous studies had shown that Internet‐based behavioral experiments generate reliable data comparable to those based on more traditional data acquisition in the lab (e.g., Zwaan & Pecher, 2012). One hundred participants participated, 50 in the Starting Small training regime and 50 in the random training regime. Fifty‐three percent of participants were male, mean age was 36.5 years (*SD* = 11.4), 15% had a high school diploma as their highest achieved education, 23% had attended college without degree, 10% had an associate's degree, 43% had a bachelor's degree, and 9% had a graduate degree (master's, doctorate, etc.). All participants completed an informed consent form prior to the start of the experiment, were from the United States, and were paid $3.00 for approximately 20 min of their time (see Buhrmester, Kwang, & Gosling, 2011).

#### 4.1.2 Materials

The learning and test sequences used in Experiment 2 were identical to those in Experiment 1b (see Online Appendix B).

#### 4.1.3 Procedure

The procedure was identical to Experiment 1b and was programmed using the QRTEngine (Barnhoorn, Haasnoot, Bocanegra, & van Steenbergen, 2015).

### 4.2 Results and discussion

**Figure 3:**
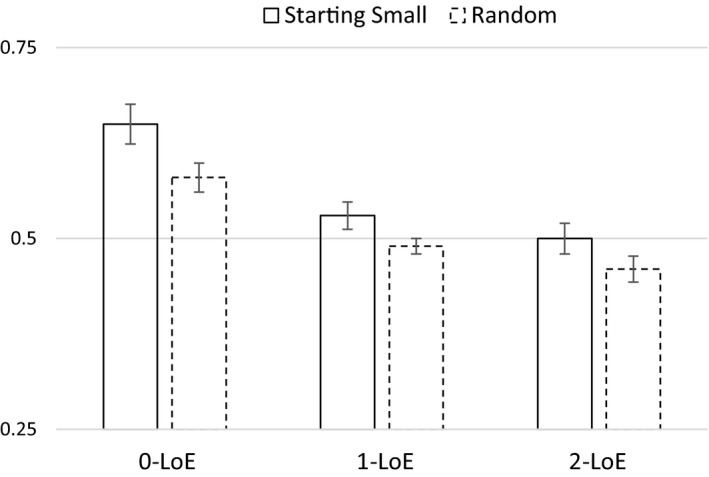
Performance for starting‐small and random‐ordering training conditions using a center‐embedded recursive grammar as a function of number of levels of embedding in the test items (Experiment 2). Error‐bars indicate *SE* of the mean.

The mean proportion of correct classification on the 50 test items was .56 for the starting‐small group (*M = *28.0 *SD* = 5.59) and .51 for the random group (*M = *25.5, *SD* = 3.52). Only the starting‐small group performed significantly above chance levels (*t*(49) = 3.82, *p *<* *.001). We performed a 3 × 2 mixed anova on mean proportions of accurate responses with Levels of Embedding (1‐LoE, 2‐LoE and 3‐LoE) as a within‐subjects factor, and Training Regime (Starting Small and Random) as a between‐subjects factor. We observed significant main effects for Levels of Embedding (*F*(2, 196) = 30.18, *p *<* *.001) and Training Regime (*F*(1, 98) = 6.88, *p = *.01), but no interaction between the two factors (*F*(2, 196) = 0.47, *p = *.63; see Fig. 3). Planned linear contrasts indicated that performance increased with decreasing numbers of levels of embeddings (*p *<* *.001). Pairwise comparisons on the factor Levels of Embeddings showed that 0‐LoE items (*M = *.61, *SD* = .17) had a higher accuracy than 1‐LoE items (*M = *.51, *SD* = .11; *p *<* *.01), and 2‐LoE items (*M = *.48, *SD* = .13; *p *<* *.01), and that 1‐LoE items had marginally significant higher accuracy than 2‐LoE items (*p = *.052).

Despite the lack of a significant interaction between Levels of Embedding and Training Regime, we performed simple‐effects analyses of LoE for Starting Small and Random conditions separately. The Starting Small condition showed a significant LoE effect (*F*(2, 98) = 16.72, *p *<* *.01), where 0‐LoE (*M = *.65, *SD* = .18) had a higher accuracy than both 1‐LoE (*M = *.53, *SD* = .13; *p *<* *.01) and 2‐LoE (*M = *.50, *SD* = .14; *p *<* *.01). The Random condition also showed a significant LoE effect (*F*(2, 98) = 13.48, *p *<* *.01), where 0‐LoE (*M = *.58, *SD* = .14) had a higher accuracy than both 1‐LoE (*M = *.49, *SD* = .08; *p *<* *.01) and 2‐LoE (*M = *.47, *SD* = .12; *p *<* *.01).

As in Experiment 1b, the results show that only when the input was presented in a staged fashion were participants showing above‐chance learning of the artificial grammar. Furthermore, the Starting Small condition outperformed the Random condition, and this effect was independent of the number of levels of embeddings in the test items. Experiment 2 replicates the starting‐small effect for center‐embedded recursive structures, in a larger and more general sample with a more diverse educational background than in Experiments 1a and 1b, which suggests that the facilitation occurs for learners with a broad range of cognitive skills.

Both Experiments 1a, 1b and 2 used recursive grammars with the same basic structure, pairing two elements of two categories A and B, having different recursive operations. Given that all pairings had equal lengths (i.e., an A with a B letter), strings with an equal number of embeddings necessarily have equal lengths in both RB and CE grammars: 0‐LoE strings have two letters, 1‐LoE strings have four letters, and 2‐LoE strings have six letters. As a result, a starting‐small ordering based on the number of LoE's correlates perfectly with ordering according to increasing length, in both RB and CE grammars. Therefore, the results of Experiments 1a, 1b and 2 are inconclusive with respect to the relative contribution of length (by staging input according to string length) and structural complexity (by staging input according to LoE). Though string length has been suggested to affect learning independently from complexity in a non‐recursive AGL study (Poletiek & Van Schijndel, 2009), previous studies on the *less is more* and *starting small* effects have not distinguished between these two contributions.

Thus, it is conceivable that staging the training input according to increasing string *length* facilitates learning. The alternative view is that reduced *complexity* during initial learning—that is gradually increasing the number of embeddings over time—is the more important factor causing the starting‐small facilitation. As we argued above, learners must first identify the basic structural pattern before they can generalize it to recursive constructions that reuse the basic pattern. A starting mall regime allows for early elaborate encoding of the basic patterns and chunks of the system, before they are encountered in more complex structures. Hence, we hypothesize that starting small helps because it gradually introduces increasingly complex structures following initial exposure to basic patterns, not because it incrementally stages the *number* of elements in the input per se.

Basic patterns underlying recursive constructions in natural language are not limited to two‐element pairings, as in Experiments 1a, 1b, and 2, but vary in length. This makes parsing even more difficult because the building blocks a sentence is made of cannot be identified on the basis of their length. In Experiment 3, we use a recursive grammar that produces strings of variable length within a given LoE. Furthermore, we compare a starting‐small training regime according to string length and a starting‐small training regime according to string complexity, to determine the relative impact of length and computational constraints when starting small. In summary, our results so far suggest a beneficial effect of starting small for recursive grammars but leaves unanswered whether the effect was due to a gradual increase in string length or in structural complexity during training. Note that the answer to this question has implications for the issue of the learnability of complex center‐embedded structures by exposure to exemplars: If the ordering constraint (incremental ordering over time) in the input can be exploited effectively to learn the structure of language, this might strengthen the possibility that human languages may be learnable from environmental information in the input (see also Christiansen & Chater, 1999, 2015; Christiansen & MacDonald, 2009; Poletiek & Chater, 2006).

## 5 Experiment 3

**Figure 4:**
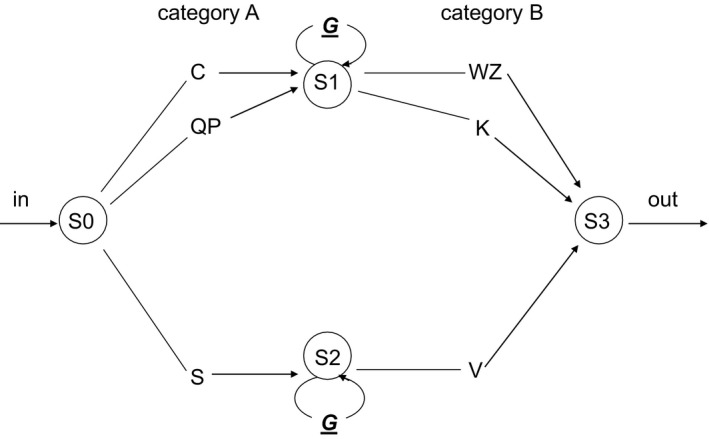
Center‐embedded grammar G3, used in Experiment 3, with exemplars varying in length for an equal number of LoE. For example, QP[CWZ]WZ (length 7) and S[CK] V (length 4) are exemplars of this grammar having both 1 LoE.

Similar to Experiments 1b and 2, a recursive center‐embedded artificial grammar was used with two categories A and B, in this case consisting of eight letters. However, the basic elements in each category (A and B) were either individual letters or bigrams. Category A elements were C, QP, S, and Category B elements were WZ, K, V. Category A elements could be paired with Category B elements from the same subset, as displayed in Fig. 4.

This resulted in a grammar *G* with the same structural characteristics as Grammar G‐CE, but having fewer legal AB pairings. In contrast to the stimuli in Experiment 1a, 1b, and 2, the basic 0‐LoE items could be two, three, or four letters long. The variation in length was realized by replacing two letters by specific bigrams (QP and WZ). This change was intended to change as little as possible the complexity of the elements per se, while still varying sentence length for equally complex (i.e., equal number of embeddings) sentences. The length of 1‐LoE items could vary in length between four and eight letters. Hence, the length of a string was not fully dependent on its recursive complexity. For example, strings with four letters could have either no (QPWZ) or one embedding (C[SV]K). Notice that LoE and length were not fully independent either, because inserting an embedding necessarily increases the length of a string. As can be seen in Fig. 4, five unique legal AB pairs (0‐loE sequences) could be generated by G (as compared to G‐CE having 18 unique legal AB pairs). In this manner, we reduced the variability in *G* to compensate for its increased complexity as compared to the grammars in Experiments 1a, 1b, and 2, caused by the variability in string length within a level of embedding, in order to make learning possible within the context of the experimental task.

### 5.1 Method

#### 5.1.1 Participants

To allow a reliable comparison between the two starting‐small regimes and one random regime, while still controlling the task performing conditions in the laboratory, we enhanced statistical power by increasing sample size. Fifty‐four students (18 in each of the three groups) from Leiden University participated, either for course credits or financial compensation (€4.50).

#### 5.1.2 Materials

Fifty grammatical sequences were generated from the grammar G, for the training set (Online Appendix C). Each exemplar was presented three times. The 50 strings were ordered (within each experimental block) based on “groups” of strings differing in LoE or length. In the structure‐based Starting Small (SS‐S) condition, the exemplars were presented successively in three consecutive groups of five 0‐LoE sequences, followed by fifteen 1‐LoE sequences, and finally thirty 2‐LoE exemplars. Within a group, the ordering of the strings was randomized and each unique sequence was presented three times (Online Appendix C). Following the same procedure, the same fifty sequences were ordered according to their length in the Starting Small according to length (SS‐L) condition. In the SS‐L condition, grouping was determined by string length. Ten groups were presented successively: two unique strings with length 2, followed by two unique strings of length 3, two strings of length 4, four strings of length 5, eleven strings of length 6, seven strings of length 7, eleven strings of length 8, eight strings of length 9, two strings of length 10, and one string of length 12 (see Online Appendix C).

Within a group, the sequences were presented randomly. As in the SS‐S condition, the unique sequences were presented three times each in a random order, with the constraint that one unique sequence could not be repeated. For groups with two strings, the strings were alternated three times. The string in the last group (one string of length 12) could of course not satisfy the non‐repetition requirement. It was repeated three times. In the Random condition, the 50 strings were presented in random ordering in one single group, each three times. No consecutive repetitions could occur in the random presentation.

The test set consisted of 25 grammatical and 25 ungrammatical strings. As in Experiment 1a, 1b, and 2, ungrammatical sequences were created by changing one element of a grammatical test sequence. The substituted element was one that was of the proper category (a B was replaced with a B element) but from an incorrect subset, hence making an incorrect pair with the corresponding A element. The positions in which the substituted elements occurred in the sequences were distributed evenly across all items (Online Appendix C). Since the present grammar G3 generated only five unique 0‐LoE sequences, these could occur in both the training and test set. The 1‐LoE and 2‐LoE test items did not occur in the training set.

#### 5.1.3 Procedure

E‐Prime presentation software was used to present the stimuli on a computer monitor. Participants were randomly assigned to one of three conditions: Starting Small‐Structure based (SS‐S), Starting Small‐length based (SS‐L), or Random. All participants were instructed that they were participating in a memory experiment. Training sequences were presented individually, for a duration of 4‐s each. In each condition, the same 50 training items were presented three times, in successive groups, for a total of 150 input exposures. Depending on condition, grouping was determined on the basis of structure (number of levels of embedding) in the SS‐S group, and on the basis of string length in the SS‐L group. In the random condition, the input was fully randomized (Online Appendix C).

After training, participants were told that they would see new letter sequences. Notice, however, that four LoE training strings were repeated in the test phase. This was not expected to affect their performance, because they judged whether the new sequences followed the same rules as the training sequences or not. Participants were required to respond as quickly and accurately as possible. Each test sequence was presented on the screen until a judgment was made.

### 5.2 Results and discussion

The mean proportion of correct classification was .63 for the Starting Small‐Structure group (*M = *31.5*, SD* = 8.5), .52 for the Starting Small‐Length group (*M* = 26.2*; SD* = 3.4), and .45 for the Random group (*M = *22.4*, SD* = 3.8). We performed a 3 × 3 mixed anova on mean proportions of accurate responses with Levels of Embedding (1‐LoE, 2‐LoE and 3‐LoE) as a within‐subjects factor, and Training Regime (Starting Small‐Structure, Starting Small‐Length, and Random) as a between‐subjects factor. Due to a violation of sphericity (Mauchly's *W = *.49, *p *<* *.001), we examined multivariate within‐subjects tests. We observed significant main‐effects for Levels of Embedding (Wilks” λ = .86, *F*(2, 50) = 4.08, *p = *.02), and Training Regime (*F*(2, 51) = 12.25, *p *<* *.001), but no interaction between the two factors (Wilks” λ = .85, *F*(4, 100) = 2.17, *p = *.08). Planned linear contrasts indicated that performance increased with decreasing numbers of levels of embeddings (*p = *.014) and increased as a function of training regime (*p *<* *.001). Helmert contrasts indicated that the average performance in the starting‐small conditions was higher than in the random condition (*p *<* *.01), and that the Starting Small‐Structure condition resulted in a higher performance than the Starting Small‐Length condition (*p *<* *.01). Pairwise comparisons on the factor Levels of Embeddings showed that 0‐LoE items (*M = *.57, *SD* = .23) had a higher accuracy than 2‐LoE items (*M = *.50, *SD *= .13; *p = *.01), and comparisons on the factor Training Regime showed that Starting Small‐Structure (*M = *.63, *SD* = .17) outperformed Starting Small‐Length (*M = *.52, *SD* = .07; *p = *.006) and Random (*M = *.45, *SD* = .08; *p *<* *.001), and Starting Small‐Length outperformed Random (*p = *.046). All other pairwise comparisons were not significant.

Despite the lack of a significant interaction between Levels of Embedding and Training Regime, we performed simple‐effects analyses of LoE for SS‐S, SS‐L and Random conditions separately. The SS‐S condition showed a significant LoE effect (*F*(2, 34) = 6.45, *p *<* *.01), where 0‐LoE (*M = *.71, *SD* = .20) had a higher accuracy than both 1‐LoE (*M = *.61, *SD* = .18; *p = *.02), and 2‐LoE (*M = *.58, *SD* = .19; *p = *.01). The SS‐L condition also showed a significant LoE effect (*F*(2, 34) = 5.47, *p = *.02), where 0‐LoE (*M = *.60, *SD* = .17) had a higher accuracy that both 1‐LoE (*M = *.486, *SD* = .09; *p = *.02), and 2‐LoE (*M = *.490, *SD* = .05; *p = *.02). The random condition did not show a LoE effect: 0‐LoE (*M = *.42, *SD* = .21) did not have a higher accuracy than both 1‐LoE (*M = *.48, *SD* = .10; *p = *.33) and 2‐LoE (*M = *.45, *SD* = .06; *p = *.57).

In addition, we also performed simple‐effects analyses of the factor Training Regime for the 0‐LoE, 1‐LoE, and 2‐LoE conditions separately. The effect of Training Regime was significant for 0‐LoE items (*F*(2, 51) = 10.18, *p *<* *.001), 1‐LoE items (*F*(2, 51) = 5.77, *p *<* *.01), and 2‐LoE items (*F*(2, 51) = 5.43, *p *<* *.01). Pairwise comparisons for 0‐LoE items showed that Starting Small‐Structure outperformed Random (*p *<* *.001), and Starting Small‐Length outperformed Random (*p = *.008), for the 1‐LoE items that Starting Small‐Structure outperformed Starting Small‐Length (*p = *.01) and outperformed Random (*p = *.01), for 2‐LoE items that Starting Small‐Structure nearly outperformed Starting Small‐Length (*p = *.07) and outperformed Random (*p = *.01), and Starting Small‐Length outperformed Random (*p = *.03). All other pairwise comparisons were not significant.

In summary, the results of Experiment 3 showed a facilitation of starting small for learning the center‐embedded grammar, especially when the training items were staged according to increasing LoE, and less so when the items were staged according to increasing string length. Overall, 0‐LoE test items were judged more accurately than 2‐LoE test items.

A possible limitation for generalizing to natural grammar learning is the presentation of full “sentences” during the learning and testing phases. Under the current experimental setup, all elements of the sentence were presented simultaneously and stayed available during processing, potentially facilitating the pattern recognition of elements that are distant from each other. Natural language processing, however, unfolds sequentially over time, and words must be bound together in real time (e.g., Christiansen & Chater, 2016). Words heard at the onset of the sentence have to be either continuously maintained or retrieved from memory, and selected for integration with upcoming words. In complex sentences with multiple embeddings, processing difficulties might rapidly increase when the words are presented sequentially rather than simultaneously.

In Experiment 4, we test whether starting small helps when the learning input and the to‐be‐judged test sentences are processed sequentially word by word, like in natural language. As in Experiment 3, we hypothesize that starting small with basic patterns first might facilitate generalization to more complex sequences that contain these basic patterns. In the simultaneously presented materials, visual pattern recognition might be involved in this learning. Alternatively, during sequential presentation of the strings, processing might involve learning to predict over time, as in natural language. Hence, the first element of a basic structure might activate prediction of the second one. Alternatively, the second element of a basic structure might act as a cue to retrieve the first one. We, therefore, performed an additional analysis testing whether performance on 0‐LoE items predicts performance on the more complex (1‐LoE and 2‐LoE) items for two starting‐small conditions and one random presentation condition.

## 6 Experiment 4

**Figure 5:**
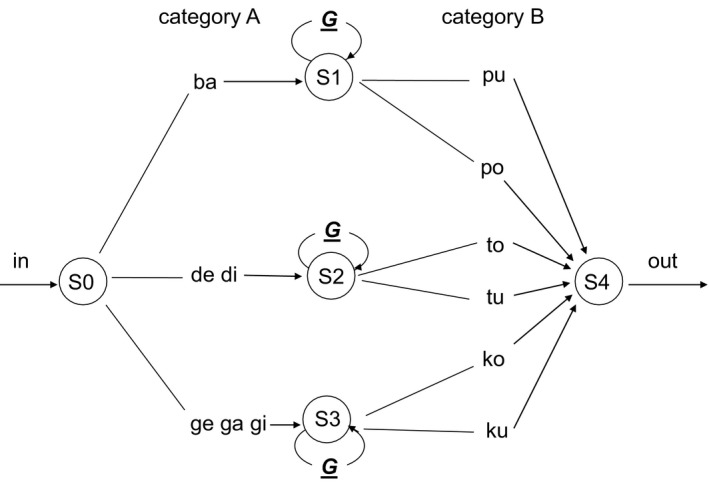
Center‐embedding grammar *G4* with two categories of elements (A‐elements and B‐elements), used in Experiment 4. A elements were one, two, or three syllables long; B elements were one syllable. Exemplars vary in length for an equal number of LoE., for example, gegagi[gegagiku]ko (length 8) and ba[[dedi]tu]po (length 5) are exemplars of this grammar having both 1‐LoE.

As in Experiments 1b, 2, and 3, a recursive center‐embedded artificial grammar was used with two element categories A and B. Since the sequential presentation was expected to make the task overall more difficult than in Experiment 3, adaptations were made to the grammar and the procedure, but which did not affect the crucial manipulations. First, consonant‐vowel (CV) syllables rather than letters were used to make the basic elements (“words”) of the language. The purpose of this was to facilitate reading, that is, pronouncing silently, and encoding the stream of elements fluently. To vary string length independently of the number of levels of embedding (as in Experiment 3), category A elements could vary in length (being one, two or three syllables long) (see Fig. 5). In all conditions, the same training and test items were used.

The two sets of syllables were categorized by their vowels and their consonants. Category A contained –a/‐e/‐i, whereas Category B contained ‐o/‐u. These phonetic cues constrained word category, and thus helped learning the categories, without affecting the positional embedding rule. As can be seen in Fig. 5, six unique legal AB pairs (0‐LoE sequences) could be generated by the grammar.

### 6.1 Method

#### 6.1.1 Participants

Forty‐three students from Leiden University participated in the study, either for course credits or financial compensation (€4.50).

#### 6.1.2 Materials

A sample of grammatical sequences was generated from grammar G4 to be used as the training set (Online Appendix D). The same 38 unique exemplars were presented. As in Experiment 3, they were presented repeatedly (114 learning items in total). In the structure‐based Starting Small (SS‐S) condition, the exemplars were presented successively in three groups: first, all six unique 0‐LoE sequences, followed by a group of 17 unique 1‐LoE sequences, and a group of 15 unique 2‐LoE exemplars (Online Appendix D). Following the same procedure, the same 38 sequences were ordered according to their length in the Starting Small according to length (SS‐L) condition, in which groups were determined by string length (varying from 2 syllables to 12 syllables). Eleven groups with unique strings were presented successively: two unique strings with length 2, followed by two unique strings of length 3, five strings of length 4, three strings of length 5, seven strings of length 6, eight strings of length 7, four strings of length 8, three strings of length 9, two strings of length 10, one string of length 11, and one string of length 12 (see Online Appendix D). Within a group, the sequences were presented randomly. In the Random condition, the 38 unique strings were presented repeatedly in random ordering in one single group. No subsequent repetitions could occur in the random presentation.

The test set was made of 22 grammatical and 22 ungrammatical strings. As in Experiments 1–3, ungrammatical sequences were created by changing one element of a grammatical test sequence. The substituted element was one that was of the proper category (a B was replaced with a B element) but from an incorrect subset, hence making an incorrect pair with the corresponding A element. The positions in which the substituted elements occurred in the sequences were distributed evenly across all items (Online Appendix D). Since the present grammar G4 generated only six unique 0‐LoE sequences, these could occur in both the training and test set. The 1‐LoE and 2‐LoE test items did not occur in the training set.

#### 6.1.3 Procedure

As in Experiment 1a, 1b, and 3, E‐Prime presentation software was used with stimuli presented on a computer monitor. Participants were randomly assigned to one of three conditions: Starting Small‐Structure based (SS‐S) (*n* = 15), Starting Small‐length based (SS‐L) (*n* = 15), or Random (*n* = 13). All 43 participants were instructed that they were participating in a memory experiment. Except for changes to the stimuli, and the sequential presentation, all procedural details were identical to Experiment 3. The syllables of a sequence were presented one at a time, in the middle of the screen each for a duration of 800 ms. After each completed sequence, a fixation cross appeared for 800 ms, after which the next sequence started. During the learning phase, participants were given two breaks of 1 minute. In the SS‐S condition, one break was given after the group with 0‐LoE items, and after the group with 2‐LoE items. In the SS‐L condition, the breaks were after the group with length‐4 items and after the group with length‐7 items. In the Random condition, the breaks were given after the 32nd and after 64th sequence, respectively.

### 6.2 Results and discussion

The mean proportion of correct classification was .65 for the Starting Small‐Structure group (mean number of correct responses is *M = *28.5, *SD* = 8.3), .61 for the Starting Small‐Length group (M = 26.7, *SD* = 4.7)*;* and .54 for the Random group (*M = *23.8, *SD* = 4.5). We performed a 3 × 3 mixed anova on mean proportions of accurate responses with Levels of Embedding (1‐LoE, 2‐LoE and 3‐LoE) as a within‐subjects factor, and Training Regime (Starting Small‐Structure, Starting Small‐Length, and Random) as a between‐subjects factor. Given that sphericity was not violated, we examined univariate within‐subjects tests. Although we observed a significant main‐effect for Levels of Embedding (*F*(2, 80) = 5.21, *p = *.007), the main‐effect for Training Regime failed to reach significance (*F*(2, 40) = 2.04, *p = *.14), as was the case for the interaction between the two factors (*F *<* *1, *p = *.56). Planned linear contrasts indicated that performance increased with decreasing numbers of levels of embeddings (*p = *.004), and increased as a function of training regime (*p = *.05). Helmert contrasts indicated that average performance in the starting‐small conditions was marginally better than in the random condition (*p = *.07), and that Starting Small‐Structure condition did not have a higher performance than Starting Small‐Length condition (*p = *.43). Pairwise comparisons on the factor Levels of Embeddings showed that 0‐LoE items (*M = *.65, *SD* = .17) had a higher accuracy than 1‐LoE items (*M = *.59, *SD* = .18; *p = *.018) and 2‐LoE items (*M = *.57, *SD* = .17; *p = *.004), and comparisons on the factor Training Regime showed that Starting Small‐Structure (*M = *.65, *SD *= .19) outperformed Random (*M = *.54, *SD* = .10; *p = *.05). All other pairwise comparisons were not significant.

Despite the lack of a significant interaction between Levels of Embedding and Training Regime, we performed simple‐effects analyses of LoE for SS‐S, SS‐L and Random conditions separately. None of the training conditions showed a significant LoE effect. In the SS‐S condition, 0‐LoE (*M = *.68, *SD* = .19) did not have a higher accuracy than both 1‐LoE (*M = *.62, *SD* = .22; *p = *.17), and 2‐LoE (*M = *.65, *SD* = .20; *p = *.31). In the SS‐L condition, 0‐LoE (*M = *.67, *SD* = .16) did not have a higher accuracy than 1‐LoE (*M = *.62, *SD* = .16; *p = *.34), but it did have a higher accuracy than 2‐LoE (*M = *.55, *SD* = .14; *p = *.02). In the random condition, 0‐LoE (*M = *.60, *SD* = .14) did not have a higher accuracy than both 1‐LoE (*M = *.52, *SD* = .14; *p = *.08) and 2‐LoE (*M = *.51, *SD* = .15; *p = *.14).

In addition, we also performed simple‐effects analyses of the factor Training Regime for the 0‐LoE, 1‐LoE, and 2‐LoE conditions separately. The effect of Training Regime was not significant for 0‐LoE and 1‐LoE items and nearly significant for 3‐LoE items (*F*(2, 40) = 2.58, *p = *.09). Pairwise comparisons for the 1‐LoE items that Starting Small‐Length nearly outperformed Random (*p = *.10), and for 2‐LoE items that Starting Small‐Structure nearly outperformed Random (*p = *.05). All other pairwise comparisons were not significant.

Overall, Experiment 4 shows a pattern of results comparable to Experiment 3, whereby performance is highest for 0‐LoE items, and performance is enhanced when the grammar is administered according a training regime that increases incrementally according to structural complexity. Also, these two effects appear to occur independently of each other.

**Figure 6:**
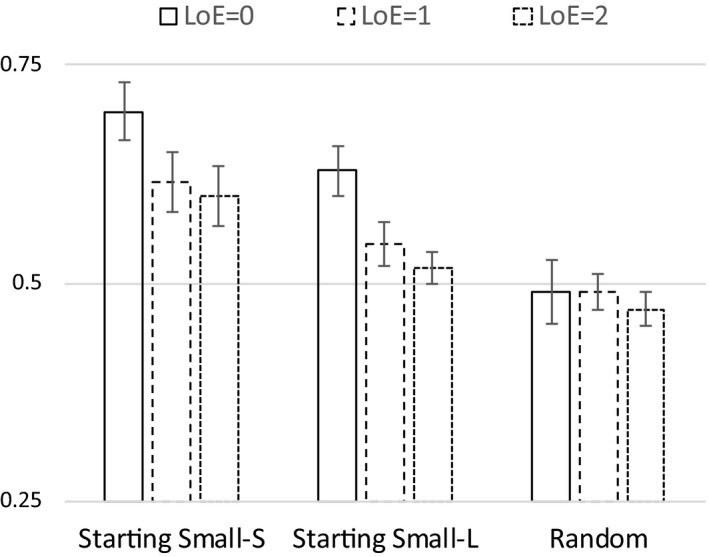
Mean performance for Starting Small‐Structure, Starting Small‐Length, and Random training conditions as a function of levels of embedding for the aggregated data of Experiments 3 and 4. Error bars indicate *SE* of the mean.

**Figure 7:**
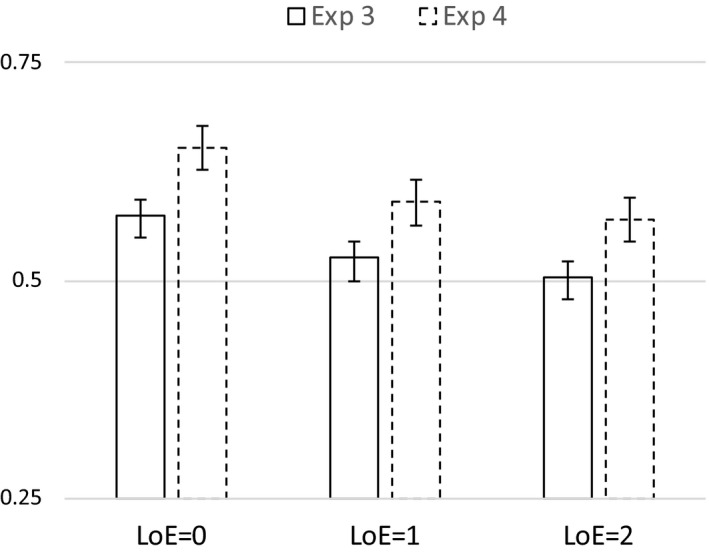
Mean performance for the three level of embedding conditions for Experiments 3 and 4. Error bars indicate within‐subject *SE* of the mean.

**Figure 8:**
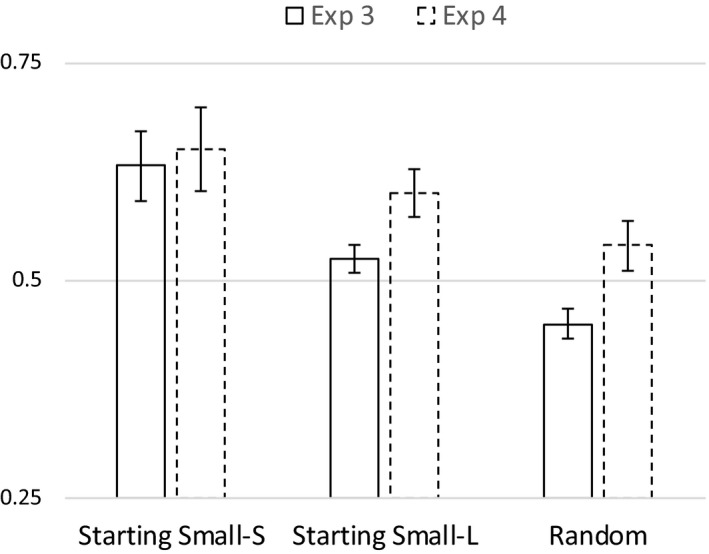
Mean performance for Starting Small‐Structure, Starting Small‐Length, and Random training conditions for Experiments 3 and 4. Error bars indicate *SE* of the mean.

In order to perform more powerful fine‐grained tests of our hypotheses, we performed additional analyses on the aggregated the data of Experiments 3 and 4 (see Fig. 6). Once more, we performed a 3 × 3 mixed anova on mean proportions of accurate responses with Levels of Embedding (1‐LoE, 2‐LoE, and 3‐LoE) as a within‐subjects factor and Training Regime (Starting Small‐Structure, Starting Small‐Length, and Random) as a between‐subjects factor. Due to a violation of sphericity (Mauchly's *W = *.83, *p *<* *.001), we examined multivariate within‐subjects tests. We observed significant main‐effects for Levels of Embedding (Wilks’ λ = .87, *F*(2, 93) = 7.05, *p *<* *.001), and Training Regime (*F*(2, 94) = 11.18, *p *<* *.001), and no interaction between the two factors (Wilks’ λ = .95, *F*(4, 186) = 1.19, *p = *.32). Planned linear contrasts indicated that performance increased with decreasing numbers of levels of embeddings (see Fig. 7; *p *<* *.001), and also increased as a function of training regime (see Fig. 8; *p *<* *.001). Helmert contrasts indicated that average performance in the starting‐small conditions was higher than in the random condition (*p *<* *.01), and that Starting Small‐Structure resulted in higher performance than Starting Small‐Length (*p = *.02).

**Figure 9:**
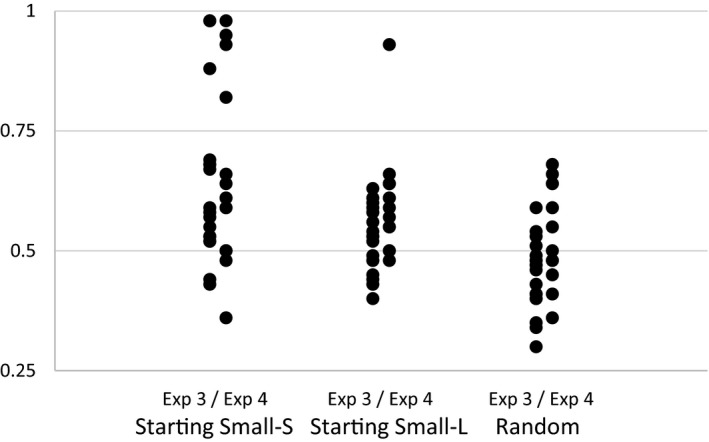
Scatterplot of accuracy scores of grammaticality judgments for the three training regimes in Experiment 3 and 4. The reference line at .50 represents chance‐level performance.

Pairwise comparisons on the factor Levels of Embeddings showed that 0‐LoE items (*M = *.61) had a higher accuracy than 1‐LoE items (*M = *.55; *p = *.009) and 2‐LoE items (*M = *.53; *p *<* *.001). Pairwise comparisons on the factor Training Regime showed that all comparisons were significant: Starting Small‐Structure (*M = *.65) outperformed Starting Small‐Length (*M = *.56; *p = *.017), and Random (*M = *.49; *p *<* *.001), and Starting Small‐Length outperformed Random (*p = *.022). Also, one‐sample *t*‐tests revealed that both Starting Small groups performed above chance level (Starting Small‐Structure group; *t*(32) = 4.61, *p *<* *.001, Starting Small‐Length group; *t*(32) = 3.69, *p *<* *.001). However, the Random group did not perform above chance level; *t* (30) = 0.76, *p = *.45. In the Starting Small‐Structure groups, six participants performed very highly, above .80 (see Fig. 9), suggesting that under this learning regime, the system could be fully learned and applied to new items.

Mean accuracies of grammaticality judgments for test items with 0‐, 1‐, and 2‐LoE for each condition were *M*_0LoE_ = .70, *SD*_0LoE_ = .20, *M*_1LoE_ = .62, *SD*_1LoE_ = .20, *M*_2LoE_ = .61, *SD*_2LoE_ = .19 for the SS_S group; *M*_0LoE_ = .63, *SD*_0LoE_ = .17, *M*_1LoE_ = .55, *SD*_1LoE_ = .14, *M*_2LoE_ = .52, *SD*_2LoE_ = .11 for the SS_L group; *M*_0LoE_ = .49, *SD*_0LoE_ = .21, *M*_1LoE_ = .50, *SD*_1LoE_ = .12, *M*_2LoE_ = .48, *SD*_2LoE_ = .11 for the random group.

Despite the lack of a significant interaction between Levels of Embedding and Training Regime, we performed simple‐effects analyses of LoE for SS‐S, SS‐L and Random conditions separately. The SS‐S condition showed a significant LoE effect (*F*(2, 64) = 6.48, *p *<* *.01), where 0‐LoE had a higher accuracy than both 1‐LoE (*p *<* *.01) and 2‐LoE (*p *<* *.01). The SS‐L condition also showed a significant LoE effect (*F*(2, 64) = 7.17, *p *<* *.01), where 0‐LoE had a higher accuracy that both 1‐LoE (*p = *.018) and 2‐LoE (*p *<* *.01). The random condition did not show an LoE effect.

In addition, we also performed simple‐effects analyses of the factor Training Regime for the 0‐LoE, 1‐LoE, and 2‐LoE conditions separately. The effect of Training Regime was significant for 0‐LoE items (*F*(2, 94) = 9.34, *p *<* *.001), 1‐LoE items (*F*(2, 94) = 4.68, *p = *.012), and 2‐LoE items (*F*(2, 94) = 7.41, *p = *.001). Pairwise comparisons for 0‐LoE items showed that Starting Small‐Structure outperformed Random (*p *<* *.001), and Starting Small‐Length outperformed Random (*p = *.006), for the 1‐LoE items that Starting Small‐Structure nearly outperformed Starting Small‐Length (*p = *.07) and outperformed Random (*p = *.003), for 2‐LoE items that Starting Small‐Structure outperformed Starting Small‐Length (*p = *.01) and Random (*p *<* *.001). All other pairwise comparisons were not significant.

**Figure 10:**
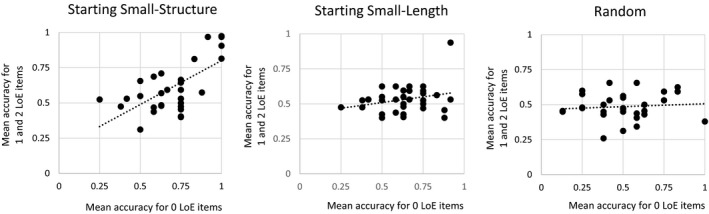
Regression models with performance for the 0‐LoE test items as predictor of performance on multiple‐embedding test items, for the Starting Small according to Structure group (left graph), for the Starting Small according to item Length group (middle graph), and for the Random group (right graph).

To test whether participants who were trained using the Starting Small‐Structure regime could better generalize their knowledge of simple structures to multiple embedded constructions, we fit regression models with performance on the 0‐LoE items as predictor of performance on multiple‐embedding test items for the aggregated data of Experiments 3 and 4 (see Fig. 10).

Performance on 0‐LoE items was a strong predictor for performance on sentences with multiple embeddings, for the Starting Small‐Structure condition only (*R* ^2^ = .44; β = .66; *p *<* *.0001). For the Starting Small‐Length and Random conditions, the beta coefficients were not significant (*R* ^2^ = .08; *p = *.12; *R* ^2^ = .01; *p = *.63, respectively; see Fig. 10). Moreover, individual differences in the group trained on a structurally growing input were larger than in the other groups. Specifically, in the Starting Small‐Structure group, 7 out of 33 participants almost perfectly learned the grammar (Mean accuracy >.80), one in the Starting Small‐Length group, and none in the Random group. Furthermore, knowledge of the basic structures could reliably predict accuracy for complex ones for Starting Small‐Structure learners only. This suggests that these learners were able to make better use of their memory for early encoded simple structures to process the more complex ones.

## 7 General discussion

Our five experiments provide unique insight into when starting small in the form of staged input may help the learner. For four recursive artificial grammars, a starting‐small training regime was compared with a random training regime. For all grammars tested, that is, a right‐branching recursive grammar, and three center‐embedded recursive grammars, the starting‐small presentation was either a necessary condition for learning or clearly facilitated learning. The starting‐small facilitation may rely on constraining two aspects of the stimulus input. First, starting small reduces the length of the sequence units to be processed initially, and second, it reduces the computational complexity during initial learning. Experiments 3 and 4 partially disentangled the effect of length from that of complexity, in order to shed light on the mechanism behind the Starting Small effect for learning recursion. A starting‐small regime based on incremental complexity was most beneficial to learning.

These data underscore the incremental character of learning complex structures by exposure to spoken exemplars (Christiansen & Chater, 2016; Lai & Poletiek, 2011, 2013; MacDonald, 2013): Learning to process embedded structures requires an initial exposure period to non‐embedded simple sentences (Lai & Poletiek, 2011). Once these simple constructions are learned, the transition to complex sentences containing the embedding structure becomes easier. Interestingly, this mechanism does not appear to work when a starting‐small regime is implemented according to length of the input. When the initially encoded sentences had reduced length but not reduced complexity, our results indicate that learning of short sequences did not affect performance on complex stimuli.

The results clearly suggest that constraining the complexity of the input effectively facilitates learning grammars that have a self‐embedding recursive structure. Our data suggest that artificial recursive structures varying from linear “additive” right‐branching structures to center‐embedding constructions involving long‐distance dependencies are learned best with a staged input of exemplars. More specifically, staging the input exemplars according to structural complexity, as opposed to length, results in the largest facilitation. Learning complex structures from exemplars seems to involve a two‐stage process where simple units are learned first, after which this knowledge is exploited to analyze more complex stimuli containing multiple units. Interestingly, the simple units learned in the first stage have been argued to crucially represent events (e.g., agent–action pairs) in the world acquired in interaction with caregivers using joint attention (Tomasello, 2003).

Our findings are in line with previous studies on the learnability of artificial complex structures that mirror natural grammar complexity. Previous studies showed that simple artificial grammars without recursion could be learned in the typical procedure we used in the present experiments (see Reber, 1993 for an overview). Moreover, this learning of simple artificial grammars has been shown to have little relationship with intelligence (Reber, Walkenfeld, & Hernstadt, 1991). Thus far, however, either no structure learning could be demonstrated (De Vries, Monaghan, Knecht, & Zwitserlood, 2008) or they demonstrated an effect only if extra‐linguistic cues in the input environment were present (De Vries, Geukes, Zwitserlood, Petersson, & Christiansen, 2012; Lai & Poletiek, 2011, 2013; Van den Bos, Christiansen, & Misyak, 2012). Elman (1993) first demonstrated the beneficial effect of starting small in a computational simulation, using a grammar similar to the ones used in this study. This study is the first to investigate and compare computational complexity (ordering according to structure) and length (ordering according to length) of starting‐small learning regimes. Overall, the present data revealed not only the beneficial effect of a structurally staged input set on learning, but also incremental processes that rely on specific aspects of what is recently learned to process new incoming input. Third, our replication of the starting‐small facilitation in a general heterogeneous population suggests the effect works independently of intelligence, as also observed in the learning of simple artificial grammars.

Interestingly, several recent AGL studies on the learnability of complex structure have used starting‐small regimes in their designs, even if it was not the focus of the study, which may have contributed to the reported results on learnability. For example, Perfors et al. (2011) proposed a Bayesian computational model for inductive learning of a complex artificial phrase grammar. The computational model was run with artificial input data based on features of child‐directed speech, where presented items incrementally increased in level of complexity. Interestingly, Bahlmann, Schubotz, and Friederici (2008) reported higher activation of Broca's area in the brain for participants who learned a recursive artificial grammar than in a group that had learned a non‐recursive artificial grammar using fMRI. Both input sets were organized in a starting‐small fashion. Also, a recent event‐related potential (ERP) study investigating the neurophysiological correlates of artificial grammar learning deliberately employed a starting‐small paradigm to ensure a high level of performance (Christiansen, Conway, & Onnis, 2012). These studies further underscore the special importance of starting small for learning grammars that contain recursive constructions.

Translating these results to more realistic situations, what do they tell us about natural language learning? To answer this question, we first need to compare the artificial grammar implemented in our study to natural language, and secondly, we need to compare the staged input implemented in the lab to the linguistic input of an actual language learner, that is, child‐directed speech. In natural language, recursive constructions occur quite frequently. In most cases, self‐referring recursive regularities occur as simple left‐ or right‐branching structures as in repeating adjectives (*the* [*big*] [*red*] [*plastic*] *ball*) and repeating sentential complements ([*Mary says*] [*that Bob thinks*] [*that Gabby saw Bill*]), respectively. Self‐embedded structures are less frequent and typically limited to a single level of embedding. Sentences with two or more levels of embedding (as in *The boy* [*the girl* [*the woman admires*] *loves*] *likes the dog*) are difficult to understand (e.g., Blaubergs & Braine, 1974; Wang, 1970; see Christiansen & MacDonald, 2009, for a review) and practically absent from spoken language (Karlsson, 2007). Interestingly, in our data, performance also decreased as sequence complexity increased. The crucial effect of exploiting memorized simple units for understanding complex ones with multiple units might reflect how natural language users use their knowledge of simple structures to chunk and organize complex structures (Christiansen & Chater, 2016; see, for example, McCauley & Christiansen, 2011, 2014, in press, for a relevant computational model).

Although biological factors appear to put prohibitive limitations on the learning of self‐embedded recursive structure (De Vries et al., 2011, 2012), the experience that a learner has with particular recursive constructions also play a key role (Christiansen & Chater, 2016; Christiansen & MacDonald, 2009; Wells, Christiansen, Race, Acheson, & MacDonald, 2009). Our results suggest that the order in which learners experience recursive structures may play an important role in how well such recursive constructions can be mastered. Specifically, starting small makes the basic structure of the system salient, and enables learners to focus on the basic structural patterns first, before they are faced with complex structures incorporating multiple instances of the basic pattern. Our results also suggest that this learning tends to display “all‐or‐nothing” mastery transitions over time. Hence, if natural language input is ordered according to a starting‐small regime, we would expect facilitation for learning recursive structures both in artificial grammars, as here, and in natural language.

This brings us to the second comparison we need to make in order to assess the external validity of the present results, which is between the two starting‐small procedures (structure‐based and length‐based) and natural child‐directed speech. If the constraints on computational capacity effectively enhance natural language learning, then the sentences a learner is exposed to should, over time, become gradually more complex rather than longer. Likewise, sentences occurring in child‐directed speech would be expected to be constrained mainly in complexity, but not necessarily in length.

Studies on early language acquisition consistently find that the language of primary caregivers includes fewer complex sentences, and sentences containing no or fewer subordinate clauses than adult speech (Brown, 1973; Pine, 1994; Tomasello, 2003). In addition, the structural complexity of early language input is reduced through the use of features marking clause boundaries, like strong variations of pitch at the end of constituents, pauses, lengthening the final syllable of words at the end of clauses, and part or whole repetitions of phrases (Cruttenden, 1994). These prosodic features facilitate the segmentation of sentences according to syntactic structure and may highlight their structural characteristics (see in an AGL context; Morgan, Meier, & Newport, 1987). This kind of prosodic segmentation in language is consistent with the manipulations in the present experiments: The features present in child‐directed speech serve to point the listener to the basic structural units first, which allow the child to generalize these basic structures to more complex structures. From this perspective, the transition from one group of learning items with *n* levels of embedding to the next group with *n* + 1 levels of embedding functionally highlights the boundaries of embedded clauses, similarly to prosodic features in natural language. Interestingly, this incremental learning mechanism is conditional upon the language allowing for the same basic pattern to be recognized at each level of complexity in sentences, and hence, for the learner, at each stage of exposure.

Although most studies on child‐directed speech also mention length constraints as a feature of early sentences (Pine, 1994), some complexity‐reducing features of child‐directed speech may actually lengthen sentences rather than shorten them, such as repetition of constituents and protracting syllables. Consistent with the present findings, this may indicate that length reduction plays a subordinate role in comparison to complexity reduction in child‐directed speech. Starting‐small orderings according to length may even misdirect the learners’ attention and point them to basic units which are structurally non‐pertinent, thus hindering their recognition of structural units in more complex sentences. These combined experimental and natural language studies lend support to the idea that reducing computational complexity (i.e., starting simple) may be more important for learning than reducing length (i.e., starting short). This is an area that warrants further research.

Besides starting small, a number of recent studies with artificial languages suggest that certain extra‐linguistic cues, also present in the natural language, substantially facilitate the learning task (Christiansen & Dale, 2001; Perruchet & Rey, 2005; Poletiek, 2002, 2006; Poletiek & Chater, 2006; Van den Bos & Poletiek, 2015; Van den Bos et al., 2012). First, the frequency distribution of a learning set may emphasize the structural properties of the underlying grammar. Poletiek and Chater (2006), Poletiek (2006), and Lai and Poletiek (2013) showed that presenting more exemplars with low complex constructions had a positive effect on learning the full grammar. Indeed, a frequency distribution that over‐represents simple compared to complex sentences may help learning (Perfors et al., 2011), and this overrepresentation has been shown to characterize child‐directed speech (Pine, 1994). Second, primacy effects may also contribute to learning in starting‐small regimes. If learners (even adult learners) are better learners at the earlier stages of exposure—as suggested by the primacy effect (Newport, Weiss, & Aslin, 2006)—a starting‐small input enables learners to acquire the basic form of a recursive construction during this “sensitive” stage (Lai & Poletiek, 2011). This possibility is supported by the findings by Lai and Poletiek (2013), who showed that the occurrence of any incidental complex sentence within a group of simple items presented in the earliest stage of exposure can already interfere with learning.

Finally, other types of cues may interact with a starting‐small effect. For example, presentation modality influences performance in AGL tasks (Conway & Christiansen, 2005; Conway et al., 2003; Saffran, 2002; see Frost, Armstrong, Siegelman, & Christiansen, 2015, for a review). Under sequential presentation conditions, humans are better at encoding and processing auditory, as compared to visual input; on the other hand, learning is also very effective when visual input is presented simultaneously rather than sequentially (Conway & Christiansen, 2009). In three of our four experiments, all elements of a stimulus were presented simultaneously. This presentation format is commonly used in AGL experimentation and may possibly have enhanced the starting‐small effect for the self‐embedded structure in Experiments 1a, 1b (Conway et al., 2003), 2, and 3. In particular, long distance dependencies may be easier to recognize when the full string is displayed visually, as compared to an auditory presentation where correct parsing depends on the retrieval of previously encountered elements. In Experiment 4, we presented the stimulus elements visually, but sequentially, to mimic the incremental memory requirements for processing recursive constructions in natural speech. We observed that facilitation due to the starting‐small learning regime was associated with reuse of early learned grammatical features in processing sentences at a later stage. However, future work must further specify the contribution of starting small under more naturalistic conditions.

Distributional characteristics of the input sample have also been shown to affect performance in AGL. Poletiek and Van Schijndel (2009) showed that learning was improved when the input sample contained sentences that were highly frequent in the full language as compared to a sample containing low frequent sentences of equal size, suggesting that high frequent sentences in the language are also very informative about the regularities underlying the grammar. Further research is needed to find out how this distributional effect relates to the time course effect of starting small.

Interestingly, the role of stimulus set characteristics, including the starting‐small effect, may provide new insights into how natural language acquisition may be accommodated within learning‐by‐exposure accounts. Our analyses suggest that a structure‐based starting‐small regime is especially advantageous for learning complex recursive structures. The starting‐small facilitation operates on the computational aspect of what has to be learned, making use of initially acquired basic chunks. Given that recursive structure is an important feature of natural language, and possibly of human cognition more broadly, the current set of results may help future studies to explore the extent to which starting small may contribute to spoken language acquisition and inductive learning more generally.

## Supporting information


**Appendix A.** Learning and test materials of Experiment 1a.
**Appendix B.** Learning and test materials of Experiment 1b.
**Appendix C.** Learning and test materials of Experiment 3.
**Appendix D.** Learning and test materials of Experiment 4.
