# Supplementary material for: Under What Conditions Can Recursion Be Learned? Effects of Starting Small in Artificial Grammar Learning of Center‐Embedded Structure
Source: Cogn Sci. 2018 Sep 27;42(8):2855–89. doi: 10.1111/cogs.12685 (PMC6585836; doi:10.1111/cogs.12685)
Supplement: Supplementary file 1 — Appendix A. Learning and test materials of Experiment 1a. Appendix B. Learning and test materials of Experiment 1b. Appendix C. Learning and test materials of Experiment 3. Appendix D. Learning and test materials of Experiment 4. [file COGS-42-2855-s001.docx]

**Appendix A**

Experiment 1: Learning exemplars of an artificial right branching recursive grammar (Figure 1a).

| 0 levels of embedding | 1 level of embedding | 2 levels of embedding |
| --- | --- | --- |
| CW | CWPT | CKMWPH |
| CK | QZMW | QWXTMK |
| QZ | MKXH | MZSHCW |
| MW | PHQK | PVQZST |
| MK | XTSV | XHQKCZ |
| PH | SVCZ | STMWXV |
| PV | QKPT | CWXHSV |
| XT | QWCK | XVCKPT |
| SH | XTMZ | SHPTQZ |
| SV | CKQW | PTSVQW |
|  | MZPV | MZPVXH |
|  | XVPH | QKPHMZ |
|  | PTCK | XTCZMK |
|  | SHMW | PVCZSH |
|  | QZST | QMMKXV |
|  | STXH | MZQWCK |
|  | CWXV | PHXTQK |
|  | MKCZ | QZXVPT |
|  | PVXT | STMWQZ |
|  | SHQZ | MKSVCW |
|  |  |  |

Experiment 1: Test materials

| Grammatical | Ungrammatical |
| --- | --- |
| CZ | **CT** |
| QW | **QH** |
| QK | **QV** |
| MZ | **MT** |
| PT | **PZ** |
| XH | **XW** |
| XV | **XK** |
| ST | **SZ** |
| PHCW | **QH**CW |
| MWQK | MW**XK** |
| XHPT | **XW**PT |
| CZXV | CZ**XW** |
| SVMZ | **CV**MZ |
| QWSH | QW**CH** |
| STPV | **SZ**PV |
| MKCZ | MK**CT** |
| MZSVCW | **XZ**SVCW |
| PVXHQK | PV**MH**QK |
| XTCZSH | XTCZ**QH** |
| QWMKPT | QWMK**PZ** |
| CKMWXV | CK**MT**XV |
| PHSTMK | **PZ**STMK |
| SVQZXT | **CV**QZXT |
| STPHQZ | ST**CH**QZ |
| XHPVCK | XHPV**SK** |
|  |  |

**Appendix B**

Experiment 2: Learning exemplars of an artificial center embedding recursive grammar (Figure 1b).

| 0 levels of embedding | 1 level of embedding | 2 levels of embedding |
| --- | --- | --- |
| CW | CPTW | CMPHWK |
| CK | QMWZ | QXMKTW |
| QZ | MXHK | MSCWHZ |
| MW | PQKH | PQSTZV |
| MK | XSVT | XQCZKH |
| PH | SCZV | SMXVWT |
| PV | QPTK | CXSVHW |
| XT | QCKW | XCPTKV |
| SH | XMZT | SPQZTH |
| SV | CQWK | PSQWVT |
|  | MPVZ | MPXHVZ |
|  | XPHV | QPMZHK |
|  | PCKT | XCMKZT |
|  | SMWH | PCSHZV |
|  | QSTZ | QMXVKM |
|  | SXHT | MQCKWZ |
|  | CXVW | PXQKTH |
|  | MCZK | QSPTVZ |
|  | PXTV | SMQZWT |
|  | SQZH | MSCWVK |

Experiment 2: Test materials

| Grammatical | Ungrammatical |
| --- | --- |
| CZ | **CT** |
| QW | **QH** |
| QK | **QV** |
| MZ | **MT** |
| PT | **PZ** |
| XH | **XW** |
| XV | **XK** |
| ST | **SZ** |
| PCWH | **Q**CW**H** |
| MQKW | M**XK**W |
| XPTH | **X**PT**W** |
| CXVZ | C**XW**Z |
| SMZV | **C**MZ**V** |
| QSHW | Q**KH**W |
| SPVT | **S**PV**Z** |
| MCZK | M**CT**K |
| MSCWVZ | **X**SCWV**Z** |
| PXQKHV | P**M**QK**H**V |
| XCSHZT | XC**QH**ZT |
| QMPTKW | QM**PZ**KW |
| CMXVWK | C**M**XV**T**K |
| PSMKTH | **P**SMKT**Z** |
| SQXTZV | **C**QXTZ**V** |
| SPQZHT | S**C**QZ**H**T |
| XPCKVH | X**P**SK**V**H |
|  |  |

**Appendix C**

Experiment 3: Learning and testing stimuli in all conditions: Starting Small according to number of levels of embedding, Starting Small according to length, and Randomly ordered. Squared brackets (not presented to participants) indicate embeddings.

| Ordering according to Levels of Embedding | | | | Ordering according to length | | | | Random ordering | | |
| --- | --- | --- | --- | --- | --- | --- | --- | --- | --- | --- |
|  | LoE | Length |  |  | LoE | Length |  |  | LoE | Length |
| QPK | 0 | 3 |  | CK | 0 | 2 |  | C[QP[CK]K]K | 2 | 7 |
| CWZ | 0 | 3 |  | SV | 0 | 2 |  | C[S[CWZ]V]WZ | 2 | 8 |
| QPWZ | 0 | 4 |  | CWZ | 0 | 3 |  | CWZ | 0 | 3 |
| CK | 0 | 2 |  | QPK | 0 | 3 |  | S[S[CK]V]V | 2 | 6 |
| SV | 0 | 2 |  | S[SV]V | 1 | 4 |  | S[QPWZ]V | 1 | 6 |
| C[CK]WZ | 1 | 5 |  | QPWZ | 0 | 4 |  | C[C[CK]WZ]K | 2 | 7 |
| C[CWZ]WZ | 1 | 6 |  | C[SV]WZ | 1 | 5 |  | QPK | 0 | 3 |
| QP[SV]WZ | 1 | 6 |  | C[CK]WZ | 1 | 5 |  | QP[SV]WZ | 1 | 6 |
| C[QPK]K | 1 | 5 |  | S[CWZ]V | 1 | 5 |  | C[C[CWZ]WZ]WZ | 2 | 9 |
| S[QPWZ]V | 1 | 6 |  | C[QPK]K | 1 | 5 |  | C[QPWZ]K | 1 | 6 |
| QP[QPK]K | 1 | 6 |  | QP[CWZ]K | 1 | 6 |  | C[QP[CWZ]K]WZ | 2 | 9 |
| S[CWZ]V | 1 | 5 |  | QP[QPK]K | 1 | 6 |  | S[S[SV]V]V | 2 | 6 |
| C[QPWZ]K | 1 | 6 |  | QP[SV]WZ | 1 | 6 |  | S[SV]V | 1 | 4 |
| QP[CK]WZ | 1 | 6 |  | S[C[SV]K]V | 2 | 6 |  | QP[CWZ]K | 1 | 5 |
| S[SV]V | 1 | 4 |  | QP[CK]WZ | 1 | 6 |  | QP[QP[QPWZ]WZ]WZ | 2 | 12 |
| C[SV]WZ | 1 | 5 |  | C[QPWZ]K | 1 | 6 |  | S[C[SV]K]V | 2 | 6 |
| QP[QPWZ]WZ | 1 | 8 |  | S[QPWZ]V | 1 | 6 |  | C[CK]WZ | 1 | 5 |
| QP[CWZ]K | 1 | 6 |  | C[C[SV]K]K | 2 | 6 |  | QP[QP[SV]WZ]K | 2 | 9 |
| QP[QPK]WZ | 1 | 7 |  | S[S[SV]V]V | 2 | 6 |  | S[QP[SV]WZ]V | 2 | 8 |
| C[QPWZ]WZ | 1 | 7 |  | S[S[CK]V]V | 2 | 6 |  | QP[C[CK]WZ]WZ | 2 | 9 |
| S[C[SV]K]V | 2 | 6 |  | C[CWZ]WZ | 1 | 6 |  | S[QP[CWZ]K]V | 2 | 8 |
| C[QP[QPK]WZ]WZ | 2 | 10 |  | C[S[SV]V]WZ | 2 | 7 |  | QP[QP[CWZ]K]K | 2 | 9 |
| S[QP[CWZ]K]V | 2 | 8 |  | QP[QPK]WZ | 1 | 7 |  | QP[CK]WZ | 1 | 6 |
| QP[C[SV]K]WZ | 2 | 8 |  | C[C[CK]WZ]K | 2 | 7 |  | S[C[CK]WZ]V | 2 | 7 |
| S[S[CK]V]V | 2 | 6 |  | C[S[QPK]V]K | 2 | 7 |  | C[CWZ]WZ | 1 | 6 |
| S[QP[SV]WZ]V | 2 | 8 |  | C[QP[CK]K]K | 2 | 7 |  | QPWZ | 0 | 4 |
| QP[S[CK]V]WZ | 2 | 8 |  | S[C[CK]WZ]V | 2 | 7 |  | C[QPWZ]WZ | 1 | 7 |
| S[C[CK]WZ]V | 2 | 7 |  | C[QPWZ]WZ | 1 | 7 |  | QP[S[QPWZ]V]K | 2 | 9 |
| QP[C[CK]WZ]WZ | 2 | 9 |  | S[C[QPWZ]K]V | 2 | 8 |  | C[SV]WZ | 1 | 5 |
| C[C[QPWZ]K]K | 2 | 8 |  | QP[S[CK]V]WZ | 2 | 8 |  | QP[C[SV]K]WZ | 2 | 8 |
| C[QP[SV]WZ]K | 2 | 8 |  | C[S[CWZ]V]WZ | 2 | 8 |  | S[CWZ]V | 1 | 5 |
| C[C[SV]K]K | 2 | 6 |  | QP[S[SV]V]WZ | 2 | 8 |  | QP[QPWZ]WZ | 1 | 8 |
| QP[S[QPWZ]V]K | 2 | 9 |  | QP[QPWZ]WZ | 1 | 8 |  | C[QP[QPK]WZ]WZ | 2 | 10 |
| QP[C[QPK]WZ]K | 2 | 9 |  | S[QP[CWZ]K]V | 2 | 8 |  | S[C[QPWZ]K]V | 2 | 8 |
| C[S[CWZ]V]WZ | 2 | 8 |  | QP[C[SV]K]WZ | 2 | 8 |  | QP[S[SV]V]WZ | 2 | 8 |
| QP[QP[CWZ]K]K | 2 | 9 |  | S[QP[SV]WZ]V | 2 | 8 |  | SV | 0 | 2 |
| S[QP[QPK]WZ]V | 2 | 9 |  | C[C[QPWZ]K]K | 2 | 8 |  | C[QP[SV]WZ]K | 2 | 8 |
| C[S[SV]V]WZ | 2 | 7 |  | C[QP[SV]WZ]K | 2 | 8 |  | QP[S[CK]V]WZ | 2 | 8 |
| S[C[QPWZ]K]V | 2 | 8 |  | S[S[QPWZ]V]V | 2 | 8 |  | CK | 0 | 2 |
| QP[QP[QPWZ]WZ]WZ | 2 | 12 |  | C[C[CWZ]WZ]WZ | 2 | 9 |  | C[C[QPWZ]WZ]WZ | 2 | 10 |
| C[C[CWZ]WZ]WZ | 2 | 9 |  | QP[QP[CWZ]K]K | 2 | 9 |  | C[QPK]K | 1 | 5 |
| C[QP[CWZ]K]WZ | 2 | 9 |  | QP[C[QPK]WZ]K | 2 | 9 |  | C[S[QPK]V]K | 2 | 7 |
| QP[S[SV]V]WZ | 2 | 8 |  | QP[S[QPWZ]V]K | 2 | 9 |  | QP[QPK]K | 1 | 5 |
| C[S[QPK]V]K | 2 | 7 |  | QP[QP[SV]WZ]K | 2 | 9 |  | C[C[QPWZ]K]K | 2 | 8 |
| C[C[QPWZ]WZ]WZ | 2 | 10 |  | C[QP[CWZ]K]WZ | 2 | 9 |  | QP[QPK]WZ | 1 | 7 |
| S[S[QPWZ]V]V | 2 | 8 |  | QP[C[CK]WZ]WZ | 2 | 9 |  | S[S[QPWZ]V]V | 2 | 8 |
| S[S[SV]V]V | 2 | 6 |  | S[QP[QPK]WZ]V | 2 | 9 |  | QP[C[QPK]WZ]K | 2 | 9 |
| QP[QP[SV]WZ]K | 2 | 9 |  | C[C[QPWZ]WZ]WZ | 2 | 10 |  | S[QP[QPK]WZ]V | 2 | 9 |
| C[QP[CK]K]K | 2 | 7 |  | C[QP[QPK]WZ]WZ | 2 | 10 |  | C[C[SV]K]K | 2 | 6 |
| C[C[CK]WZ]K | 2 | 7 |  | QP[QP[QPWZ]WZ]WZ | 2 | 12 |  | C[S[SV]V]WZ | 2 | 7 |

Experiment 3: Test items with ungrammatical elements printed bold. Squared brackets were not presented to participants

| Grammatical | Ungrammatical |
| --- | --- |
| SV | C**V** |
| CWZ | S**K** |
| QPK | QP**V** |
| QPWZ | S**WZ** |
| C[CK]K | C[C**V**]K |
| C[CWZ]K | C[SV]**V** |
| C[QPK]WZ | S[CK]**K** |
| C[SV]K | C[CWZ]**V** |
| QP[CK]K | QP[C**V**]K |
| QP[CWZ]WZ | QP[S**K**]K |
| QP[QPWZ]K | S[QP**V**]V |
| QP[SV]K | C[QP**V**]WZ |
| S[CK]V | QP[CWZ]**V** |
| S[QPK]V | QP[QP**V**]K |
| C[C[CWZ]K]K | C[C[CWZ]K]**V** |
| C[C[QPK]K]K | C[C[QPK]**V**]K |
| C[S[CK]V]WZ | C[S[CK]**K**]WZ |
| C[S[SV]V]K | C[S[S**WZ**]V]K |
| QP[C[CWZ]WZ]WZ | QP[C[SV]**V**]K |
| QP[C[SV]K]K | QP[S[SV]V]**V** |
| QP[QP[QPWZ]WZ]K | S[C[QPK]K]**K** |
| QP[QP[SV]WZ]WZ | S[QP[S**WZ**]K]V |
| QP[S[SV]V]K | QP[C[CWZ]WZ]**V** |
| S[C[QPK]K]V | QP[QP[SV]**V**]WZ |
| S[QP[SV]K]V | QP[QP[QPWZ]WZ]**V** |
|  |  |

**Appendix D**

Experiment 4: Learning and testing stimuli in all conditions: Starting Small according to number of levels of embedding, Starting Small according to length, and Randomly ordered. Squared brackets (not presented to participants) indicate embeddings.

| Ordering according to Levels of Embedding | | | Ordering according to length | | | Random ordering | | |
| --- | --- | --- | --- | --- | --- | --- | --- | --- |
|  | LoE | Length |  | LoE | Length |  | LoE | Length |
| bapo | 0 | 2 | bapo | 0 | 2 | gegagi[bapu]ku | 1 | 6 |
| bapu | 0 | 2 | bapu | 0 | 2 | dedi[bapo]to | 1 | 5 |
| dedito | 0 | 3 | dedito | 0 | 3 | ba[bapu]po | 1 | 4 |
| deditu | 0 | 3 | deditu | 0 | 3 | gegagiko | 0 | 4 |
| gegagiko | 0 | 4 | gegagiko | 0 | 4 | ba[dedi[bapo]to]pu | 2 | 7 |
| gegagiku | 0 | 4 | gegagiku | 0 | 4 | ba[ba[bapu]po]pu | 2 | 6 |
| ba[bapo]po | 1 | 4 | ba[bapo]po | 1 | 4 | dedi[gegagiku]to | 1 | 7 |
| ba[bapu]po | 1 | 4 | ba[bapu]po | 1 | 4 | bapo | 0 | 2 |
| ba[bapo]pu | 1 | 4 | ba[bapo]pu | 1 | 4 | gegagi[gegagi[bapo]ko]ko | 2 | 10 |
| ba[deditu]po | 1 | 5 | ba[deditu]po | 1 | 5 | ba[gegagi[deditu]ku]po | 2 | 9 |
| dedi[bapo]to | 1 | 5 | dedi[bapo]to | 1 | 5 | dedi[gegagi[gegagiku]ko]to | 2 | 11 |
| ba[deditu]pu | 1 | 5 | ba[deditu]pu | 1 | 5 | gegagi[gegagi[gegagiko]ku]ko | 2 | 12 |
| gegagi[bapu]ku | 1 | 6 | gegagi[bapu]ku | 1 | 6 | gegagi[dedito]ku | 1 | 7 |
| gegagi[bapo]ko | 1 | 6 | gegagi[bapo]ko | 1 | 6 | gegagi[deditu]ku | 1 | 7 |
| dedi[dedito]tu | 1 | 6 | dedi[dedito]tu | 1 | 6 | dedi[gegagiko]tu | 1 | 7 |
| ba[gegagiku]po | 1 | 6 | ba[gegagiku]po | 1 | 6 | gegagi[deditu]ko | 1 | 7 |
| gegagi[deditu]ku | 1 | 7 | ba[ba[bapu]po]pu | 2 | 6 | dedi[dedi[gegagiku]to]tu | 2 | 10 |
| dedi[gegagiku]to | 1 | 7 | ba[ba[bapo]po]po | 2 | 6 | gegagi[dedi[bapu]tu]ko | 2 | 9 |
| dedi[gegagiko]tu | 1 | 7 | ba[ba[bapo]pu]pu | 2 | 6 | ba[dedi[gegagiku]to]pu | 2 | 9 |
| gegagi[dedito]ku | 1 | 7 | gegagi[deditu]ku | 1 | 7 | ba[ba[bapo]pu]pu | 2 | 6 |
| gegagi[deditu]ko | 1 | 7 | dedi[gegagiku]to | 1 | 7 | ba[ba[deditu]po]po | 2 | 7 |
| gegagi[gegagiku]ku | 1 | 8 | dedi[gegagiko]tu | 1 | 7 | ba[ba[gegagiku]pu]pu | 2 | 8 |
| gegagi[gegagiku]ko | 1 | 8 | gegagi[dedito]ku | 1 | 7 | gegagi[gegagiku]ko | 1 | 8 |
| ba[ba[bapu]po]pu | 2 | 6 | gegagi[deditu]ko | 1 | 7 | ba[gegagiku]po | 1 | 6 |
| ba[ba[bapo]po]po | 2 | 6 | ba[dedi[bapo]to]pu | 2 | 7 | bapu | 0 | 2 |
| ba[ba[bapo]pu]pu | 2 | 6 | ba[ba[deditu]po]po | 2 | 7 | gegagiku | 0 | 4 |
| ba[dedi[bapo]to]pu | 2 | 7 | dedi[ba[bapo]pu]tu | 2 | 7 | ba[bapo]pu | 1 | 4 |
| ba[ba[deditu]po]po | 2 | 7 | gegagi[gegagiku]ku | 1 | 8 | ba[deditu]pu | 1 | 5 |
| dedi[ba[bapo]pu]tu | 2 | 7 | gegagi[gegagiku]ko | 1 | 8 | gegagi[gegagiku]ku | 1 | 8 |
| ba[ba[gegagiko]po]pu | 2 | 8 | ba[ba[gegagiko]po]pu | 2 | 8 | ba[ba[gegagiko]po]pu | 2 | 8 |
| ba[ba[gegagiku]pu]pu | 2 | 8 | ba[ba[gegagiku]pu]pu | 2 | 8 | dedito | 0 | 3 |
| gegagi[dedi[bapu]tu]ko | 2 | 9 | gegagi[dedi[bapu]tu]ko | 2 | 9 | ba[bapo]po | 1 | 4 |
| ba[gegagi[deditu]ku]po | 2 | 9 | ba[gegagi[deditu]ku]po | 2 | 9 | gegagi[bapo]ko | 1 | 6 |
| ba[dedi[gegagiku]to]pu | 2 | 9 | ba[dedi[gegagiku]to]pu | 2 | 9 | deditu | 0 | 3 |
| dedi[dedi[gegagiku]to]tu | 2 | 10 | dedi[dedi[gegagiku]to]tu | 2 | 10 | ba[deditu]po | 1 | 5 |
| gegagi[gegagi[bapo]ko]ko | 2 | 10 | gegagi[gegagi[bapo]ko]ko | 2 | 10 | dedi[dedito]tu | 1 | 6 |
| dedi[gegagi[gegagiku]ko]to | 2 | 11 | dedi[gegagi[gegagiku]ko]to | 2 | 11 | ba[ba[bapo]po]po | 2 | 6 |
| gegagi[gegagi[gegagiko]ku]ko | 2 | 12 | gegagi[gegagi[gegagiko]ku]ko | 2 | 12 | dedi[ba[bapo]pu]tu | 2 | 7 |

Experiment 4: Test items with ungrammatical elements printed bold. Squared brackets were not presented to participants

| Grammatical | Ungrammatical |
| --- | --- |
| gegagiko | ba**tu** |
| bapo | gegagi**pu** |
| dedito | dedi**po** |
| deditu | ba**ku** |
| bapu | dedi**ko** |
| gegagiku | gegagi**to** |
| gegagi[gegagiko]ku | ba[gegagiku]**tu** |
| dedi[deditu]to | ba[gegagi**pu**]po |
| gegagi[bapo]ku | dedi[bapo]**ku** |
| ba[dedito]po | dedi[gegagi**to**]tu |
| dedi[dedito]to | gegagi[bapu]**to** |
| ba[bapu]pu | gegagi[dedi**po**]ku |
| gegagi[gegagiko]ko | gegagi[deditu]**to** |
| ba[gegagiku]pu | ba[dedito]**tu** |
| dedi[ba[bapu]po]to | gegagi[ba[bapo]**ko**]ku |
| ba[gegagi[dedito]ku]po | gegagi[dedi[gegagi**pu**]to]ku |
| gegagi[dedi[dedito]to]ko | gegagi[dedi[bapu]tu]**to** |
| ba[ba[gegagiko]pu]pu | ba[gegagi[bapo]**tu**]po |
| ba[gegagi[gegagiko]ku]po | ba[gegagi[dedi**pu**]ko]po |
| dedi[gegagi[bapo]ko]to | gegagi[dedi[deditu]tu]**po** |
| ba[dedi[deditu]to]pu | gegagi[gegagi[gegagiku]**tu**]ko |
| ba[ba[deditu]pu]pu | ba[ba[gegagi**pu**]po]pu |
